# Customization of Ethylene Glycol (EG)‐Induced BmoR‐Based Biosensor for the Directed Evolution of PET Degrading Enzymes

**DOI:** 10.1002/advs.202413205

**Published:** 2025-02-10

**Authors:** Min Li, Zhenya Chen, Wuyuan Zhang, Tong Wu, Qingsheng Qi, Yi‐Xin Huo

**Affiliations:** ^1^ Department of Gastroenterology Aerospace Center Hospital College of Life Science Beijing Institute of Technology No. 5 South Zhongguancun Street, Haidian District Beijing 100081 China; ^2^ Tangshan Research Institute Beijing Institute of Technology No. 57, South Jianshe Road, Lubei District Tangshan Hebei 063000 China; ^3^ Center for Future Foods Muyuan Laboratory Zhengzhou Henan 450016 China; ^4^ State Key Laboratory of Microbial Technology Shandong University Qingdao Shandong 266237 China

**Keywords:** biosensors, directed evolution, high‐throughput screening, PET biodegradation, value‐added bioconversion

## Abstract

The immense volume of plastic waste poses continuous threats to the ecosystem and human health. Despite substantial efforts to enhance the catalytic activity, robustness, expression, and tolerance of plastic‐degrading enzymes, the lack of high‐throughput screening (HTS) tools hinders efficient enzyme engineering for industrial applications. Herein, we develop a novel fluorescence‐based HTS tool for evolving polyethylene terephthalate (PET) degrading enzymes by constructing an engineered BmoR‐based biosensor targeting the PET breakdown product, ethylene glycol (EG). The EG‐responsive biosensors, with notably enhanced dynamic range and operation range, are customized by fluorescence‐activated cell sorting (FACS)‐assisted transcription factor engineering. The ingeniously designed SUMO‐MHETase‐FastPETase (SMF) chimera successfully addresses the functional soluble expression of MHETase in *Escherichia coli* and mitigates the inhibitory effect of mono‐(2‐hydroxyethyl) terephthalic acid (MHET) intermediate commonly observed with PETase alone. The obtained SM^M3^F mutant demonstrates 1.59‐fold higher terephthalic acid (TPA) production, with a 1.18‐fold decrease in *K*_m_, a 1.29‐fold increase in *V*_max_, and a 1.52‐fold increase in *k*_cat_/*K*_m_, indicating stronger affinity and catalytic activity toward MHET. Furthermore, the SM^M3^F crude extract depolymerizes 5 g L^−1^ bis‐(2‐hydroxyethyl) terephthalic acid (BHET) into TPA completely at 37 °C within 10 h, which is then directedly converted into value‐added protocatechuic acid (PCA) (997.16 mg L^−1^) and gallic acid (GA) (411.69 mg L^−1^) at 30 °C, establishing an eco‐friendly ‘PET‐BHET‐MHET‐TPA‐PCA‐GA’ upcycling route. This study provides a valuable HTS tool for screening large‐scale PET and MHET hydrolases candidates or metagenomic libraries, and propels the complete biodegradation and upcycling of PET waste.

## Graphical abstract

PET degrading enzymes are evolved employing a novel fluorescence high‐throughput screening tool via a performance‐improved ethylene glycol (EG)‐induced BmoR‐based biosensor modified by TF engineering. PET waste is completely depolymerized into its basic units at moderate temperatures. The PET degradation product is then directly bioconverted into high‐value‐added chemicals through the established open‐loop upcycling pathways in microbial cells.

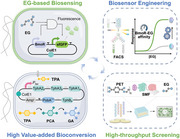

## 1 Introduction

Polyethylene terephthalate (PET) is extensively utilized in automotive, building and construction, electrical and electronics, industrial, and machinery, with a market value of $26.99 billion in 2024 and projected to reach $36.61 billion by 2029.^[^ ^1^ ^]^ However, the enormous consumption and low recovery rates have caused severe environmental damage.^[^ ^2^, ^3^ ^]^ More seriously, an increasing number of studies have reported the detection of microplastics in human tissues, raising concerns about potential health risks.^[^ ^4^ ^]^ Traditional recycling methods, both mechanical and chemical, face multiple substantial limitations.^[^ ^5^, ^6^ ^]^ Recent years have seen a notable increase in interest in plastic biodegradation. Enzymatic catalysis breaks down PET into environmentally benign products, contributing to a circular PET‐based bioeconomy.^[^ ^7^ ^]^

**Figure 1:**
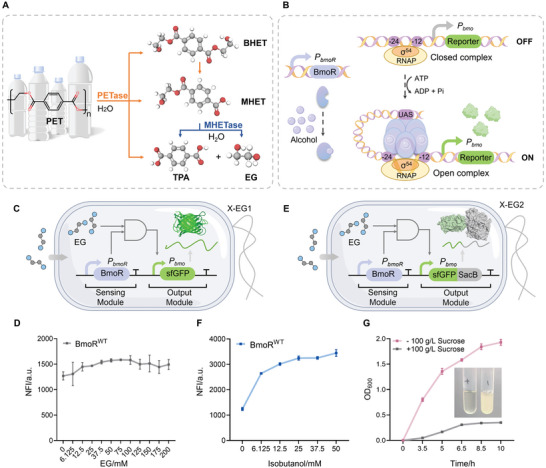
Concept of a biosensor‐derived HTS tool based on EG detection. A) PETase‐MHETase dual‐enzyme system for PET biodegradation. B) Regulation mechanism and C) composition of the BmoR‐based biosensor. *P_bmoR_*, RBS, BmoR, and terminator form the sensing module. *P_bmo_*, RBS, reporter, and terminator form the output module. D) Dose‐response curve of the BmoR^WT^‐based biosensor (pEG1) to EG. E) Composition of the BmoR‐based biosensor with negative‐selection function. F) Dose‐response curve of the BmoR^WT^‐based biosensor with negative‐selection function (pEG2) to isobutanol. G) Growth curves of the X‐EG2 strain in sucrose containing‐/free‐ medium with 10 mM isobutanol and cell growth status after 10 h. Values and error bars reflect the mean ± SD of three biological replicates (*n* = 3).

In recent decades, plenty of PET degrading enzymes have been discovered and engineered, belonging to classes of cutinases,^[^ ^8^, ^9^, ^10^, ^11^, ^12^, ^13^ ^]^ lipases,^[^ ^14^ ^]^ and esterases.^[^ ^15^ ^]^ Cutinases are predominant and show the greatest promise due to their flat, large active pockets.^[^ ^7^, ^16^ ^]^ PET degrading enzymes are characterized as thermophilic (e.g., LCC,^[^ ^9^, ^10^ ^]^ HiC,^[^ ^8^ ^]^ Cut190,^[^ ^11^ ^]^ *Tf*Cut2^[^ ^13^ ^]^) and mesophilic enzymes (e.g., *Is*PETase,^[^ ^12^ ^]^ *Is*MHETase,^[^ ^12^ ^]^ Fsc,^[^ ^8^ ^]^ BsEstB^[^ ^14^ ^]^). In enzyme engineering, considerable attention has been given to LCC and *Is*PETase as key template enzymes. Thermophilic LCC has been engineered into variants such as ICCG^[^ ^9^ ^]^ and LCC‐A2,^[^ ^10^ ^]^ which function near the glass transition temperature of PET, making them as promising and attractive candidates for industrial‐scale PET biorecycling and uprecycling, particularly for macro‐ and meso‐plastics. Mesophilic enzymatic depolymerization is emerging as a promising strategy with advantages for the in situ treatment of PET waste in environmental settings,^[^ ^17^ ^]^ especially micro‐ or nano‐plastics in soil and water. *Is*PETase and *Is*MHETase, identified from *Ideonella sakaiensis* 201‐F6, are particularly notable for their specific PET depolymerization activity at moderate temperatures,^[^ ^12^ ^]^ and have been the focus of extensive engineering efforts to improve their properties for greater industrial applicability. *Is*PETase hydrolyzes PET into mainly mono‐(2‐hydroxyethyl) terephthalic acid (MHET), with small amounts of bis‐(2‐hydroxyethyl) terephthalic acid (BHET), terephthalic acid (TPA) and ethylene glycol (EG).^[^ ^12^ ^]^ MHETase converts MHET back into the basic units of PET,^[^ ^12^ ^]^ namely TPA and EG (**Figure** **1**A). Recent advancements in *Is*PETase engineering have generated mutants with enhanced depolymerization efficiency and thermostability, such as DuraPETase,^[^ ^18^ ^]^ ThermoPETase,^[^ ^19^ ^]^ FAST‐PETase,^[^ ^20^ ^]^ HotPETase,^[^ ^21^ ^]^ DepoPETase.^[^ ^22^ ^]^ However, these PET hydrolases still show weak catalytic activity toward high‐crystallinity PET and intermediate MHET,^[^ ^23^ ^]^ resulting in inadequate degradation and reduced purity of degradation products. MHET competitively binds to the active sites of PET hydrolases, inhibiting their effects on polymers or oligo‐polymers.^[^ ^24^, ^25^, ^26^ ^]^ Therefore, MHET conversion is a limiting factor of the overall depolymerization process.^[^ ^26^, ^27^ ^]^ Multiple studies have demonstrated that transforming MHET benefits PET depolymerization efficiency and monomer proportion compared to employing PET hydrolases alone.^[^ ^15^, ^23^, ^28^, ^29^ ^]^ Engineering PET and MHET hydrolases or mining novel hydrolases is the definite way to promote efficient and effective PET biodegradation and enable downstream applications.

Directed evolution represents a powerful approach to enhancing enzyme properties, especially for beneficial distal sites that are challenging to identify employing in silico technologies.^[^ ^30^ ^]^ However, the low‐throughput and high‐cost of the existing function‐based screening tools targeting depolymerization products limit the efficiency of the ‘design‐build‐test‐learn (DBTL)’ cycle in enzyme engineering and mining. Recently, fluorescent assays in 96‐well plates were established based on PET oligomer model substrates^[^ ^22^, ^31^ ^]^ or synthetic fluorogenic probes.^[^ ^32^ ^]^ Very recently, the production of 2‐hydroxyterephthalate (TPA‐OH) formatted from bis (2‐hydroxyethyl) 2‐hydroxyterephthalate (BHET‐OH) was used to evaluate the PETase activity. The screened DepoPETase showed a higher T_m_ value by 23.3  °C and enhanced depolymerization efficiency.^[^ ^22^ ^]^ However, these assays based on PET structural analogs did not reflect the hydrolysis of actual PET polymers,^[^ ^32^ ^]^ and were unsuitable for MHETase evolution due to either extensive or minimal hydrolysis on MHET.^[^ ^22^, ^31^ ^]^ To date, there have been limited studies on high‐throughput screening (HTS) tools for the direct detection of complete depolymerization products, TPA or EG, for screening PET and MHET hydrolases candidates and metagenome libraries.

Transcription factor (TF)‐based biosensors (TFBs) offer a high‐throughput platform by coupling input signal intensity to output signal intensity in a dose‐dependent manner.^[^ ^33^ ^]^ Input signals are either biochemical molecules (e.g., metabolites,^[^ ^34^, ^35^, ^36^ ^]^ contaminants,^[^ ^37^ ^]^ biomarkers^[^ ^38^ ^]^) or physical factors (e.g., light,^[^ ^39^ ^]^ temperature^[^ ^40^ ^]^). TFBs have proven to be versatile tools for detecting numerous molecules in HTS of overproducers or superior biological blocks,^[^ ^35^, ^40^ ^]^ as well as in environmental detection^[^ ^37^ ^]^ and disease diagnosis.^[^ ^38^ ^]^ Developing biosensor‐based tools for characterizing PET hydrolases and detecting PET breakdown products, like TPA and EG, holds promise for advancing enzymatic plastic degradation. To date, the only reported TPA‐based sensors rely on the TF‐based genetical circuits (including TphR^[^ ^41^, ^42^, ^43^ ^]^ and XylS^[^ ^44^ ^]^), or reductase‐LuxAB‐coupled chemiluminescence reporting system,^[^ ^45^ ^]^ showing high‐throughput potential in the plastic hydrolases activity screening. Researchers have constructed TphR‐based biosensors in *Acinetobacter baylyi*,^[^ ^42^ ^]^ *Comamonas thiooxidans*,^[^ ^43^ ^]^ and *Pseudomonas putida* ^[^ ^41^ ^]^ to recognizing TPA, while Li et al.^[^ ^44^ ^]^ engineered the XylS^W88C‐L224Q^‐based biosensor in *Escherichia coli* (*E. coli*) for TPA detection. However, the cellular uptake of TPA and its analogs, such as MHET and BHET, impedes accurate in vivo detection through TPA‐induced biosensors. The metabolic background and physiological state of cells, along with the transient nature of bioluminescence signals, limit the application of LuxAB biosensors. With TPA detection in living cells, these tools have yet to be validated with real PET depolymerase libraries. Additionally, there are currently no reports on the EG inducible biosensor, showing its development value in EG‐based plastic degradation, particularly for PET.

BmoR, a promiscuous alcohol‐dependent TF, was identified from *Thauera butanivorans*.^[^ ^46^ ^]^ Upon sensing alcohol molecules, it undergoes a conformational change to form the activated BmoR‐alcohol complex that exhibits enhanced affinity for the upstream activating sequence (UAS) of the BmoR‐regulated promoter, *P_bmo_*.^[^ ^47^ ^]^ The σ^54^ factor specifically binds to the −24 (GG) and −12 (TGC) regions of *P_bmo_*, forming an RNA polymerase holoenzyme (Eσ^54^) with the polymerase core.^[^ ^48^ ^]^ However, it is unable to open the DNA double‐strand without the assistance of BmoR‐alcohol hexamer.^[^ ^49^, ^50^ ^]^ The BmoR‐alcohol hexamer interacts with the σ^54^ factor with DNA looping,^[^ ^47^, ^48^ ^]^ which facilitates DNA unwinding and restructuring the Eσ^54^‐DNA complex from a closed to an open conformation using the energy generated by ATP hydrolysis associated with the hexamer.^[^ ^51^, ^52^ ^]^ This structural rearrangement activates the *P_bmo_* transcription, thereby producing analyzable biosignals (Figure 1B), typically fluorescence signals. Our previous studies have established BmoR‐based biosensors for HTS higher alcohol overproducers in *E. coli* ^[^ ^35^, ^53^, ^54^ ^]^ and dynamic regulating the microbial cell factory.^[^ ^36^ ^]^ The promiscuous specificity of BmoR is a challenge for accurate detection. Alternatively, it offers the possibility of customizing other specific alcohol‐induced variants. EG, an alcohol molecule with two hydroxyl groups, is a potential ligand for BmoR. Tailor‐making a biosensor for evaluating EG production could serve as a valuable tool for rapid screening of EG‐based plastic hydrolases candidates or metagenome libraries.

**Scheme 1:**
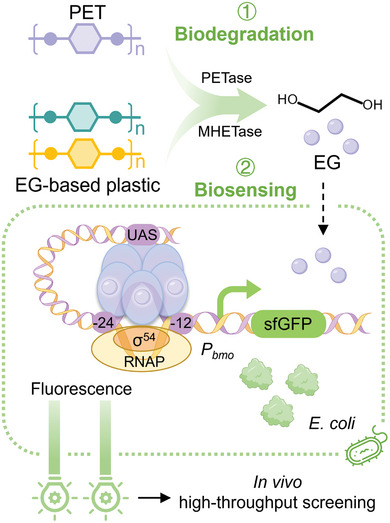
Fluorometric detection of EG using engineered BmoR‐Based whole‐cell biosensors. The biodegradation of PET by PETase and MHETase releases monomeric molecules including EG and TPA (not shown). EG can be sensed by the engineered transcription factor BmoR, which activates *P_bmo_*‐regulated transcription. The resulting fluorescence signal is used to evaluate the activity of enzyme candidates.

In this study, a novel biosensor‐derived visual HTS strategy was deployed for the directed evolution of MHETase by evaluating EG production (**Scheme** **1**). First, we engineered BmoR to achieve high sensitivity to EG, obtaining satisfactory BmoR mutants through a ‘fluorescence‐activated cell sorting (FACS) primary screening – SacB negative selection – 96 deep‐well plate (DWP) verification’ three‐step screening procedure. Subsequently, we explored the solubilizing effect of various fusion proteins on MHETase expression at different transcriptional rates, promoting soluble expression in *E. coli* BL21(DE3) by tagging soluble proteins to double‐terminals. Following that, the superior SM^M3^F chimera with enhanced MHET hydrolysis efficiency was identified through the biosensor‐based HTS tool. In silico multi‐analysis provided deeper insights into the structure‐function relationship of MHETase. Lastly, we demonstrated the potential of SM^M3^F in converting PET waste into high‐value‐added chemicals, highlighting its applicability for complete PET depolymerization and upcycling.

## 2 Results and Discussion

### 2.1 Constructing and Characterizing a BmoR‐Based Biosensor

Given that BmoR was sensitive to mono‐alcohols, we supposed that it might also respond to diols, such as ethylene glycol, the end‐product of PET degradation. To test this, we constructed an alcohol‐induced whole‐cell biosensor by integrating the sensing and output modules (Figure 1C). BmoR was expressed under the control of the constitutive promoter, *P_bmoR_*. The transcription intensity of the reporter was modulated by the paired σ^54^‐dependent promoter *P_bmo_* and varied with alcohol concentrations. Yeom et al.^[^ ^55^ ^]^ and Zhao et al.^[^ ^56^ ^]^ suggested that replacing the reporter with a super‐folder green fluorescent protein (sfGFP) was beneficial to improve the sensitivity and dynamic range of TFBs. Following this suggestion, we constructed the *P_bmoR_*‐BmoR‐*P_bmo_*‐sfGFP biosensing system (pEG1) in *E. coli* XL10‐Gold (X‐EG1) (Figure 1C). Here, sfGFP served as a visual indicator for evaluating EG production. We initially evaluated the response of wild‐type BmoR (BmoR^WT^) to EG through dose‐response testing. The strain X‐EG1 grew robustly across the range of 0–200 mM EG without growth inhibition, especially at high EG concentrations compared to isobutanol. The dose‐response curve of the BmoR^WT^‐based biosensor demonstrated a gradual increase in normalized fluorescence intensity (NFI) with increasing EG concentrations, with a maximum NFI (NFI_max_ = 1584.67 ± 16.95) at 75 mM EG (Figure 1D). However, the BmoR^WT^‐based biosensor encountered several challenges: 1) pronounced leakage expression in the absence of EG (NFI_0_ = 1268.57 ± 80.05); 2) a limited dynamic range of only 1.25‐fold; and 3) low sensitivity with minor differences observed between EG concentrations. These suboptimal performance parameters restricted its application potential for HTS of PET and MHET hydrolases. It was therefore necessary to engineer the BmoR‐based biosensor to achieve dual improvements of low background and high responsiveness to EG.

### 2.2 Constructing a BmoR‐Based Biosensor with Negative‐Selection Function

Given the modularity and evolvability of TFs, TF engineering is a common strategy to fine‐tune the biosensing behavior.^[^ ^57^ ^]^ BmoR possesses a ligand‐binding domain (LBD) and a DNA‐binding domain (DBD), which influence signal recognizing and processing, respectively. Structural modification to BmoR could impact BmoR‐ligand affinity and BmoR‐DNA affinity, as well as its physicochemical properties, ultimately improving its sensing performance. In this study, we employed random mutagenesis to evolve BmoR, aiming to generate mutants with superior responsiveness to EG.

To eliminate BmoR mutants with severe leaky expression from the library, we used the SacB protein as a negative selection marker, fused with sfGFP by a glycine‐serine linker (GGSGGGSGG). The resulting *P_bmoR_*‐BmoR‐*P_bmo_*‐sfGFP‐SacB biosensing system (pEG2) was developed (Figure 1E). The *sacB* gene encodes levansucrase, catalyzing the synthesis of levan from sucrose. Yet the accumulation of levan in *E. coli* causes cytotoxicity and cell death.^[^ ^58^ ^]^ Due to BmoR^WT^ representing a high affinity for isobutanol,^[^ ^53^ ^]^ isobutanol was selected as the ligand to test the negative‐selection function of SacB. The dose‐response of the BmoR^WT^‐based biosensor to isobutanol presented a marked fluorescence response in the range of 0–50 mM isobutanol (Figure 1F). The NFI_0_ was 1241.06 ± 57.76 without isobutanol, and the NFI_max_ was 3446.53 ± 129.57 with 50 mM isobutanol, resulting in a dynamic range of 2.78‐fold. To further validate the negative selection, the growth curves of strain X‐EG2 were measured in LB medium supplemented with 100 µg mL^−1^ ampicillin (LB/Amp_100_) and 10 mM isobutanol, with or without 100 g L^−1^ sucrose. After 10 h cultivation, X‐EG2 cells exhibited poor growth in the sucrose‐containing medium due to isobutanol‐induced *sacB* transcription, whereas they grew vigorously in the sucrose‐free medium (Figure 1G). This indicated that SacB was effective as a negative selection marker. Cells with server SacB leaky expression in the absence of EG were inhibited in the sucrose‐containing medium, enabling the selection of superior mutants with low basal interference and high signal‐to‐noise ratio.

### 2.3 Directed Evolution of Transcription Factor BmoR

**Figure 2:**
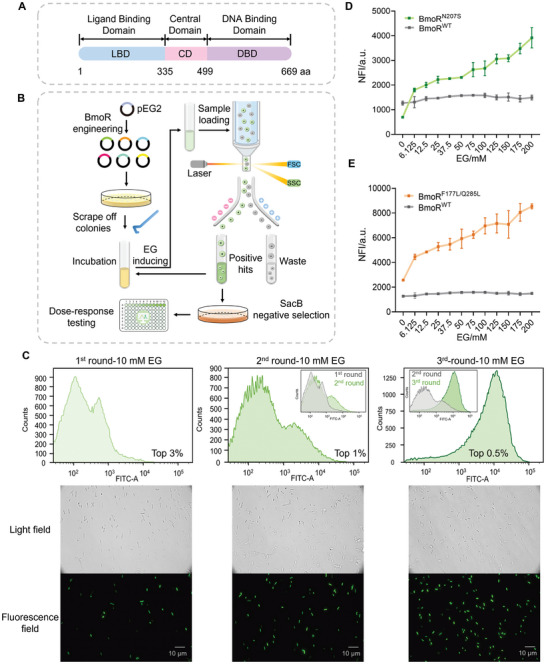
Features of BmoR and its directed evolution. A) The sequence features of BmoR. B) Schematic outline of the directed evolution of BmoR. C) Fluorescence distribution and photographs of BmoR mutation libraries in each round of FACS. D,E) Dose‐response curves of BmoR^N207S^‐/BmoR^F177L/Q285L^‐based biosensors to EG within the range of 0–200 mM in XL10‐Gold. Values and error bars reflect the mean ± SD of three biological replicates (*n* = 3).

Based on sequence and structure analysis, the σ^54^‐dependent TF BmoR possesses three characteristic domains of the bacterial enhancer binding protein (bEBP) family:^[^ ^35^, ^47^ ^]^ LBD, central domain (CD), and DBD (**Figure** **2**A). The N‐terminal LBD (1–335 aa) is responsible for sensing and capturing alcohol molecules, thereby determining specificity and overall activity.^[^ ^35^ ^]^ The LBD exhibits high sequence variability, making it a common target for engineering efforts.^[^ ^54^, ^59^ ^]^ The CD (336–499 aa) facilitates TF oligomerization and ATP hydrolysis and is highly sequentially conserved.^[^ ^51^ ^]^ Mutations in the CD are likely to impair the response activity of BmoR.^[^ ^35^ ^]^ The C‐terminal DBD (500–669 aa) features a helix‐turn‐helix structure, enabling BmoR binding to the UAS of *P_bmo_* through its specific sequence and spatial configuration.^[^ ^60^ ^]^ To develop an EG‐induced BmoR‐based biosensor with enhanced dynamic range and sensitivity, we constructed a diverse random mutation library using ep‐PCR, targeting both the LBD and the entire open reading frame of BmoR to modulate the BmoR‐EG and BmoR‐DNA affinities. Libraries with different mutation frequencies were generated by altering the final Mn^2+^ concentration (0.125, 0.25, and 0.5 mM) in the ep‐PCR reaction systems, while maintaining a constant Mg^2+^ concentration of 4 mM. The mutation frequencies at final Mn^2+^concentrations of 0.125, 0.25, and 0.5 mM were 3–5‰, 3–7‰, and 5−10‰, respectively. We subsequently selected a lower mutation frequency of 0.125 mM final Mn^2+^ concentration for further studies. The purified ep‐PCR products were subcloned into the pEG2 backbone and transformed into XL10‐Gold cells.

A three‐step screening strategy of ‘FACS primary screening ‐ SacB negative selection ‐ 96‐DWP verification’ was implemented to screen the BmoR mutation library (a library capacity of ≈50 000) with high genetic diversity (Figure 2B). In the first step, FACS was employed to sort cells based on fluorescence signals at the single‐cell level, considerably expediting the directed evolution of BmoR. The sensitivity of BmoR mutants was assessed by linking BmoR activation to sfGFP expression at a final concentration of 10 mM EG, and hyperfluorescent cells were sorted by flow cytometry. The first round of FACS collected the top 3% of cells, totaling 20 000 cells. The second round collected the top 1% of cells, totaling 10 000 cells. The third round collected the top 0.5% of cells, totaling 5 000 cells. The fluorescence distribution from the first to the third round of FACS, along with microscopy images shown in Figure 2C, demonstrated a clear shift toward higher fluorescence and a substantial proportion increase in hyperfluorescent cells. Following the third round of FACS, 1/10th of the collected cells (200 µL) were spread onto an LB/Amp_100_ agar plate containing 100 g L^−1^ sucrose and incubated overnight at 37 °C. Sanger sequencing of ten randomly picked single colonies revealed mutations in BmoR across all ten cells, with a total of five genotypes, indicating that mutants highly responsive to EG were selectively enriched after three rounds of FACS. These five BmoR mutants (BmoR^Mut^) were subsequently assessed in 96‐DWP to generate dose‐response curves for further analysis. Both the mutants BmoR^N207S^ and BmoR^F177L/Q285L^ exhibited satisfactory dose‐response curves to EG. Besides, BmoR^F177L/Q285L^ carried synonymous mutations at residues A76 and R120. Obviously, the NFI values of the two BmoR^Mut^‐based biosensors increased markedly compared to the BmoR^WT^‐based biosensor within the range of 0–200 mM EG, with both showing markedly enhanced dynamic range and sensitivity (Figure 2D,E). The BmoR^N207S^‐based biosensor (pEG3) and BmoR^F177L/Q285L^‐based biosensor (pEG4) reached their maximum fluorescence intensity at 200 mMm EG, with NFI_max_ values of 3917.18 ± 411.36 and 8536.09 ± 201.78, respectively, representing 2.47‐ and 5.39‐fold increases over the NFI_max_ of the BmoR^WT^‐based biosensor. The dynamic ranges were 5.64‐ and 3.32‐fold, respectively, showing 4.51‐ and 2.66‐fold increases over that of the BmoR^WT^‐based biosensor. We attempted to replace the high‐copy origin ColE1 with the medium‐copy origin p15A to further decrease basal fluorescence, however, causing a loss of detectability (Figure S1A,B, Supporting Information). This likely occurred because the reduced abundance of BmoR at the replication level diminished the number of activated BmoR‐EG complexes and then lowered the transcriptional initiation efficiency of *P_bmo_*. The indirect impact of reporter abundance at the transcription level and its direct impact at the replication level, ultimately impaired the sensing performance. This highlights that the abundance of TFs and reporters strongly influences the TFB performance, and suggests that altering the plasmid copy number is a feasible strategy for optimizing TFBs. To further optimize the dose‐response behavior of BmoR‐based biosensors, including minimizing the basal response, and maximizing sensitivity and dynamic range, the design of experiments (DoE) methodology offers a promising approach.^[^ ^61^ ^]^ This method enables systematically mapping combinations of genetic elements (e.g., origin, promoter, RBS, TF binding sites), omitting the need for multiple iterative rounds of permutating different combinations.^[^ ^61^ ^]^ Compared to the BmoR^F177L/Q285L^‐based biosensor, the BmoR^N207S^‐based biosensor exhibited a higher dynamic range (5.64‐fold) and a lower leaky expression level (NFI_0_ = 694.66 ± 15.17). Consequently, the BmoR^N207S^‐based biosensor was *de facto* selected for HTS of highly active PET and MHET hydrolases. The comparison of the sensing performance of the BmoR^WT^ and BmoR^Mut^ based‐biosensors was summarized in **Table** **1**.

**Table 1:** BmoR‐based biosensor performance parameters and standard deviations.

TF	NFI_max_	Leaky expression [NFI_0_]	Operation range	Dynamic range
BmoR^WT^	1584.67 ± 16.95	1268.57 ± 80.05	0–75 mM	1.25‐fold
BmoR^N207S^	3917.18 ± 411.36	694.66 ± 15.17	0–200 mM	5.64‐fold
BmoR^F177L/Q285L^	8536.09 ± 201.78	2574.43 ± 77.27	0–200 mM	3.32‐fold

### 2.4 In silico Analysis of Performance Improved BmoR Mutants

**Figure 3:**
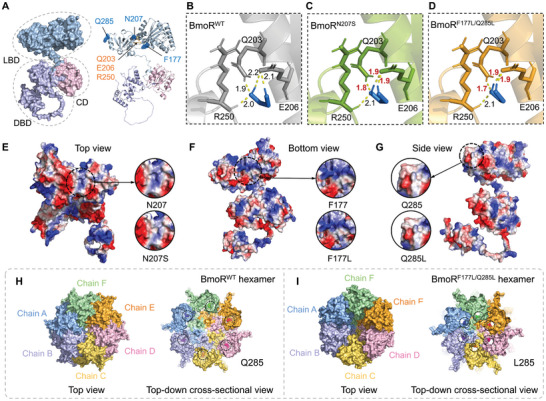
In silico analysis of BmoR and its mutants. A) The predicted structure of BmoR^WT^ by AlphaFold 2,^[^ ^62^ ^]^ is shown in both surface and cartoon representations, with the positions of residues N207, F177, and Q285 indicated. Molecular docking results of B) BmoR^WT^, C) BmoR^N207S,^ and D) BmoR^F177L/Q285L^ with EG. EG was shown as a blue stick. Yellow dotted line: hydrogen bond. Local surface electrostatic potential comparisons near the residues E) N207S, and F) F177L, and G) Q285L. Positive electrostatic potential regions were shown in blue, neutral electrostatic regions in white, and negative electrostatic potential regions in red. Top view and top‐down cross‐sectional view of H) BmoR^WT^ hexamer and I) BmoR^F177L/Q285L^ hexamer, shown in surface representation. H) Q285 residues and I) L285 residues were highlighted within black dotted circles, and depicted as spheres with a darker color. The hexamer structures were modeled by AlphaFold 3.^[^ ^63^ ^]^

EG was sensed by the activator BmoR, and the resulting BmoR‐EG complex showed increased affinity to the UAS of *P_bm_*_o_, thereby upregulating the transcription of the sfGFP‐SacB fusion protein. As anticipated, an increased fluorescence signal was observed using the two BmoR^Mut^‐based biosensors in the presence of EG (Figure 2D,E). To illustrate the molecular mechanisms of the improved sensing performance, we conducted molecular docking simulations and structural analysis. The detailed interactions of BmoR^WT^, BmoR^N207S^, and BmoR^F177L/Q285L^ with ethylene glycol (EG) were examined. In the BmoR^WT^‐EG complex, EG interacted with residues Q203, E206, and R250, with binding distances of 1.9, 2.1, 2.2, and 2.0 Å, respectively, resulting in a binding energy of −2.22 kcal mol^−1^ (**Figure** **3**B). The N207S mutation, situated on an α‐helix, reduced the local positive electrostatic potential (Figure 3A,E). This mutation brought EG closer to residues Q203 (1.8 Å) and E206 (1.9, 1.9 Å), with a slightly improved binding energy of −2.28 kcal mol^−1^ (Figure 3C). Similarly, the F177L mutation in the loop region and the Q285L mutation in the α‐helix of BmoR^F177L/Q285L^ caused minor reductions in local positive and negative electrostatic potentials, respectively (Figure 3A,F,G). Residues involved in the binding of EG to the BmoR^F177L/Q285L^ mutant included Q203 (1.7 Å), E206 (1.9, 1.9 Å), and R250 (2.1 Å), with an improved binding energy of −2.43 kcal mol^−1^ (Figure 3D). These mutations positioned EG closer to key residues Q203 and E206, enhancing the binding affinity. The closer proximity and reduced hydrogen bond distances likely increased the affinity of BmoR^N207S^ and BmoR^F177L/Q285L^ for EG. The reduction of local surface electrostatic potential might favor the stabilization of the overall conformation (Figure 3E–G). TFs typically function as homodimers or multimers, with BmoR forming a hexamer. The oligomerized TFs boost the affinity and specificity toward their specific binding sites.^[^ ^47^ ^]^ AlphaFold 3 modeling results suggested that the BmoR^F177L/Q285L^ hexamer adopted a more compact conformation compared to the BmoR^WT^ hexamer, particularly within the CDs and their near quaternary structures (Figure 3H,I). The Q285 residues in the BmoR^WT^ hexamer were more spatially separated than the L285 residues in the BmoR^F177L/Q285L^ hexamer (Figure 3H,I), indicating that the mutation resulted in a tighter oligomerization assembly. Further analysis of interchain interactions between the CDs of adjacent chains revealed an increase in noncovalent interactions, with 13 hydrogen bonds in the BmoR^F177L/Q285L^ compared to 11 in the BmoR^WT^ (Figure S2A,B, Supporting Information). These favorable structural improvements involving CDs facilitated σ^54^‐dependent BmoR oligomerization and contributed to a more stable hexameric assembly and enhanced structural integrity, ultimately favoring ATP hydrolysis and transcription activation.^[^ ^47^ ^]^ These changes collectively improve the activation efficiency of the BmoR‐EG complexes on *P_bmo_*. The directed evolution approach, which does not require an in‐depth understanding of structure‐function relationships, has proven effective in engineering TFs like BmoR to improve biosensing performance.

### 2.5 Promoting Functional Soluble Expression of MHETsae

MHET remains largely residual when using PET hydrolases alone,^[^ ^21^ ^]^ not only exerting an inhibitory effect but also diminishing the bioeconomic benefits of downstream applications. MHETase was regarded as the key enzyme to promote complete PET depolymerization.^[^ ^64^ ^]^ However, direct expression of a codon‐optimized MHETase gene in pET28a(+) vectors under *P_T7_* (pLM1) or *P_tac_* (pLM2) promoters resulted in nearly all MHETase being expressed in inclusion bodies in the cytoplasm (Figure S3A, Supporting Information). MHETase contained five disulfide bonds (residues 35–76, 208–513, 287–304, 324–332, and 561–583 in this study; Figure S4A, Supporting Information), crucial for proper folding and function.^[^ ^65^ ^]^ Disulfide bond formation required multiple biomolecules and an oxidative environment,^[^ ^65^, ^66^ ^]^ possibly explaining the folding difficulties in *E. coli* BL21(DE3). Additionally, the solubility prediction of MHETase using the Protein‐Sol website showed a low‐scaled solubility value of 0.211, below the population average of 0.45, indicating its inherent solubility challenge in *E. coli*. Its poor solubility substantially hindered its potential as a biocatalyst.

**Figure 4:**
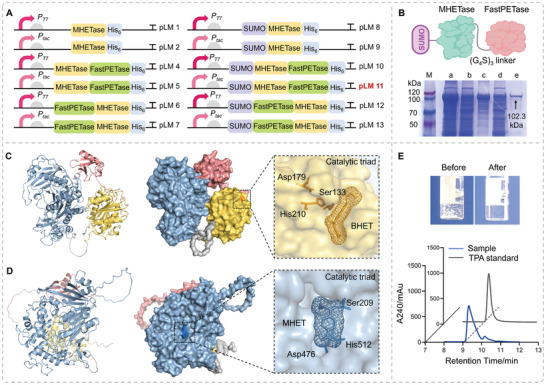
Soluble expression of MHETsae and functional verification. A) Fusion expression forms of MHETase. B) Diagram of SUMO‐MHETsae‐FastPETase fusion protein and the SDS‐PAGE result for pLM11. The gel image was a part of Figure S3D (Supporting Information) (the fifth black dotted box), with the lanes and marker (M) positions preserved. Lane M: marker, lane a: total protein, lane b: supernatant, lane c: sediment, lane d: flow‐through, lane e: purification. Docking analysis of SMF fusion protein structure predicted by AlhahFold 2 with C) BHET and D) MHET. SUMO tag: salmon. (G_4_S)_3_ linker and 6 × His tag: gray. MHETase: blue, FastPETase: yellow‐orange. Note that the low confidence level of the loop structure between SUMO and MHETase was predicted by AlphaFold 2,^[^ ^62^ ^]^ while the rest of the major structure was reliable. E) Comparison of MHET suspension before and after degradation, and UHPLC elution curves of TPA standard and MHET‐degraded solutions.

We explored various fusion protein forms at different transcription rates to achieve soluble intracellular expression of MHETase for further directed evolution (**Figure** **4**A). We attempted to enable highly soluble proteins to drive the soluble expression of poorly soluble proteins. The machine learning‐predicted FAST‐PETase (FastPETase), known for its enhanced thermal stability and activity, was currently the most efficient mesophilic PETase mutant at 50 °C.^[^ ^20^ ^]^ FastPETase showed high solubility and was predominantly expressed in soluble form in *E. coli* BL21(DE3) cells (Figure S3B, Supporting Information). The structure of FastPETase contained only two disulfide bonds (Figure S4B, Supporting Information), with a predicted scaled solubility value of 0.409 using the Protein‐Sol website, 1.94‐fold higher than that of MHETase. Fusion expression with FastPETase enabled the degradation of PET to TPA and EG abundantly promising and attractive. Both the MHETase‐FastPETase and FastPETase‐MHETase chimeras were constructed using a flexible (G_4_S)_3_ glycine‐serine linker and controlled by *P_T7_* or *P_tac_* promoters (pLM4−pLM7). Additionally, solubilization tags were commonly fused to proteins to increase their solubility and stability, such as MBP, TrxA, GST, SUMO, and NusA.^[^ ^67^ ^]^ We also employed a SUMO tag at the N‐terminal of MHETase to generate the SUMO‐MHETase fusion protein (pLM8−pLM9). However, initial attempts at the six MHETase fusion proteins with a single‐terminal solubilization tag (pLM4−pLM9) did not achieve the desired soluble expression, resulting in inclusion bodies or not expressed (Figure S3C,D, Supporting Information). We then explored dual solubilization tags by adding an N‐terminal SUMO tag to both MHETase‐FastPETase and FastPETase‐MHETase (pLM10−pLM13). Interestingly, SDS‐PAGE results revealed that adding highly soluble proteins to the double terminals of MHETase, namely SUMO‐MHETase‐FastPETase (SMF) fusion protein, under a lower transcription rate (*P_tac_*) achieved optimal soluble expression (pLM11) (Figure 4B; S3D, Supporting Information), whereas other three designs resulted in marginal solubility (Figure S3D, Supporting Information).

Subsequently, we conducted structural modeling via AlphaFold 2^[^ ^62^ ^]^ to investigate the influence of fusion expression forms on catalytic mechanisms. Comparative analysis revealed that the major structures of each component within the SMF fusion protein closely resembled their original structures (Figure S5A–F, Supporting Information). Docking analysis (Figure 4,D) with MHET and BHET substrates demonstrated that the active sites of MHETase and FastPETase within the SMF chimera closely resembled those of their standalone components, maintaining the same Ser‐His‐Asp catalytic triad.^[^ ^20^, ^28^, ^68^ ^]^ The grid boxes were specially adjusted to encompass the entire fusion protein, enabling free substrate conformation and positional changes. The overall structural similarity and consistency of the catalytic mechanism suggested that the double‐terminal solubilization tags did not adversely affect the native structural and catalytic properties. Additionally, an in vitro degradation assay confirmed the functional activity of the SMF fusion protein. When purified SMF protein was incubated with MHET powder at 37 °C (Tris‐HCl, pH 8.0), the solution was clarified, indicating near‐complete degradation. UHPLC analysis confirmed the presence of TPA in the solution (Figure 4E).

These results demonstrated that the use of double‐terminal solubilization tags facilitated the proper folding of MHETase in an active form. The fused MHETase:FastPETase dual‐enzyme system improved PET depolymerization efficiency by alleviating competitive MHET inhibition and promoting enzyme proximity effects, without requiring immobilization technology, thus promoting enzyme synergy compared to free enzymes. The incorporation of double‐terminal tags effectively illustrates that highly soluble proteins can function as classic solubility tags, promoting appropriate protein folding. Overall, our study provides valuable insights into the soluble expression of challenging proteins. The strategy of ‘leveraging highly soluble proteins to drive the soluble expression of poorly soluble proteins’ offers a practical solution for overcoming the low or non‐expression of heterologous proteins in a commonly used strain *E. coli* BL21(DE3).

### 2.6 Directed Evolution of MHETase via the BmoR‐Based Biosensor

**Figure 5:**
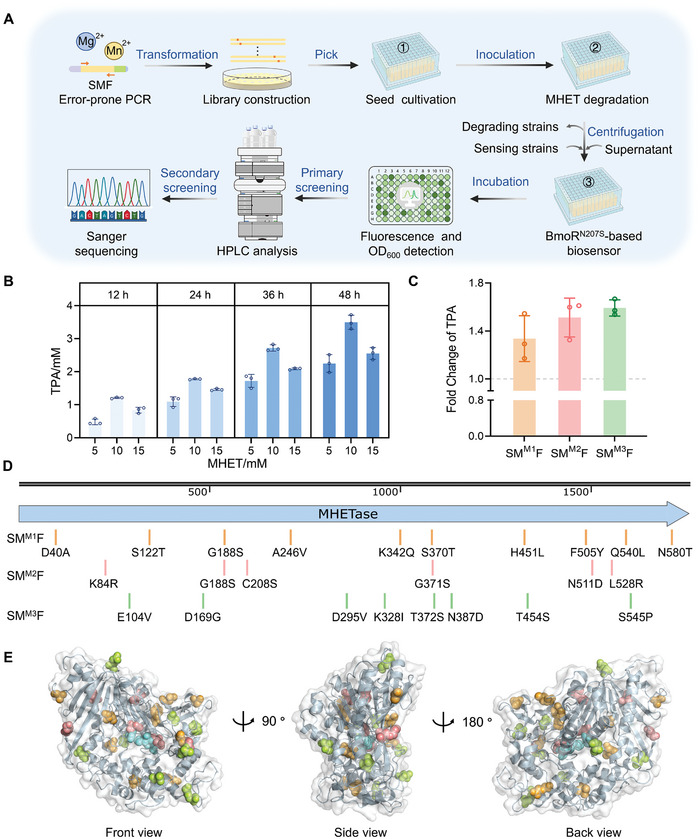
HTS of the MHETase mutant library. A) Workflow for EG inducible biosensor‐assisted HTS of MHETase library. B) TPA production at different MHET initial concentrations. C) Fold change of TPA production of mutants SM^M1^F, SM^M2^F, and SM^M3^F compared with SM^WT^F. D) Sequence and E) spatial distribution of mutation residues. Residues were shown in spheres, and proteins were shown in cartoons. Orange: SM^M1^F mutation residues. Salmon: SM^M2^F mutation residues. Limon: SM^M3^F mutation residues. Aquamarine: catalytic triad. Values and error bars reflect the mean ± SD of three biological replicates (*n* = 3).

EG, the hydrolysate of MHET, was the specific ligand for BmoR^N207S^, initiating the transcription of sfGFP upon being recognized. The BmoR^N207S^‐based biosensor connected EG sensing with fluorescence readouts, enabling the use of the microplate reader or microfluidics techniques instead of low‐throughput, costly liquid chromatography techniques. The performance of biosensors is strongly influenced by the abundance of TFs and reporters, as well as by the host genome.^[^ ^69^ ^]^ The context‐dependent activity of biological parts can eventually cause unwanted changes in the input–output response of a circuit, thereby disrupting the designed function.^[^ ^70^ ^]^ To ensure plasmid compatibility and avoid the context effects from direct and indirect interactions with cellular resources and components, we considered maintaining the existing contextual settings of the sensing strains in the HTS protocol design (host, key plasmid parts). As a result, we adopted a two‐step strategy that temporally and spatially separated the degradation and biosensing processes across two hosts to produce stable fluorescence outputs, rather than using a two‐plasmid co‐transformation approach. The epPCR product of MHETase was cloned into the pLM11 plasmid backbone to generate the random mutation library in the *E. coli* BL21(DE3) host. First, MHET was degraded to produce EG by incubating with different degradation strain variants in LB/Kan_50_ medium. After IPTG induction at 16 °C and MHET degradation at 37 °C, the mutation library cells were removed by centrifugation. Subsequently, the supernatant was incubated with the sensing strain *E. coli* X‐EG7 to evaluate EG production. To enable both strains to grow under the same antibiotic resistance conditions, the resistance gene of the BmoR^N207S^‐based biosensor plasmid was changed from *Amp^r^* (pEG3) to *Kan^r^* (pEG7). The diagram illustrating the biosensor‐based primary HTS in the 96‐well DWP format and UHPLC secondary screening is shown in **Figure** **5**A. Compared with HTS methods based on fluorogenic PET oligomer model substrates (BHET‐OH,^[^ ^22^ ^]^ mUPETs,^[^ ^31^ ^]^ and fluorescein dibenzoate^[^ ^32^ ^]^), our HTS assay offered simplicity and reality on actual PET substrate. Before screening, the optimal initial MHET concentration that would generate the highest amount of EG under consistent protein conditions was determined. As shown in Figure 5B, MHET was continuously degraded into TPA and EG overtime at all concentrations, with the highest TPA production at 10 mM MHET. After 48 h of bioconversion at 37 °C, the B‐LM11 strain produced 2.25, 3.50, and 2.55 mM TPA from initial MHET concentrations of 5, 10 and 15 mM, respectively. Therefore, 10 mM MHET was selected as the substrate concentration for the HTS system.

The MHETase mutation libraries were constructed at final Mn^2^⁺ concentrations of 0.125, 0.25, and 0.5 mM, resulting in mutation frequencies of 2–7‰, 3–8‰, 9–15‰, respectively. The libraries with low and medium mutation frequencies generated at 0.125 and 0.25 mM Mn^2+^ were subjected to being screened. MHETase libraries were conjugated with FastPETase, and improved conjugative systems were identified following the two‐step screening strategy. The NFI value of the stain expressing the wild‐type SMF (SM^WT^F) protein was used as a positive control in each plate. Almost all individual colonies (≈2000) were screened in the primary screening step based on fluorescence readouts. Subsequently, the resulting 95 selections were subjected to secondary screening using UHPLC to confirm their activity. Ultimately, the top three chimera mutants, SM^M1^F (D40A, S122T, G188S, A246V, K342Q, S370T, H451L, F505Y, Q540L, and N580T), SM^M2^F (K84R, G188S, C208S, G371S, N511D, and L528R) and SM^M3^F (E104V, D169G, D295V, K328I, T372S, N387D, T454S, and S545P) were identified after one round of determinate evolution, with 1.34‐, 1.55‐ and 1.59‐fold enhanced TPA production, respectively (Figure 5C). The sequences of wild‐type MHETase (MHETase^WT^) and three MHETase mutants were aligned, and mutation sites were marked (Figure 5D). The mutation sites were randomly distributed across the sequence without obvious hotspot regions. The spatial distribution of these residues showed that the majority were distributed around the enzyme structure, distal from the catalytic pocket (Figure 5E). The distal mutations harnessed the allosteric network of noncovalent interactions, leading to a redistribution of the relative stabilities of the conformational states to stabilize high‐energy states or favor states more competent for higher hydrolytic activity, thereby producing a population shift in the conformational landscape of MHETase and translating into increased activity.^[^ ^71^ ^]^ These results demonstrated the feasibility of the biosensor‐based HTS system for MHETase. We anticipated that the activity of MHETase could be further enhanced by several rounds of iterative evolution facilitated by our fluorescent HTS tool.

Subsequently, single‐point mutations were constructed for the 23 mutated residues in MHETase mutants to analyze the contribution of each mutation to the degradation activity. The TPA production fold change of all single‐point mutants compared to SM^WT^F was shown in Figure S6 (Supporting Information). Mutants SM^E104V^F, SM^G188S^F, SM^T372S^F, and SM^L528R^F showed 1.17‐, 1.31‐, 1.32‐, and 1.35‐fold increases over SM^WT^F, respectively. These four catalytic efficiency‐enhancing single‐point mutations were then combined to create eleven combinatorial mutants, including double, triple, and quadruple mutants. Only the SM^G188S/T372S^F mutant showed a 1.06‐fold increase in TPA production compared to SM^WT^F, while all the other combinatorial mutants had a decreased TPA production. This indicated that a simple rational design was highly challenging to obtain more desirable mutants due to ignoring synergistic interactions between distal mutations. The underlying mechanisms of beneficial distal mutations in MHETase variants are subject to further rationalization through reconstructing the conformational landscapes and analyzing the enzyme conformational dynamics of MHETase^WT^ and its variants.^[^ ^71^ ^]^

### 2.7 Enzymatic Assay, In silico Analysis and Visual Degradation of MHETase

**Figure 6:**
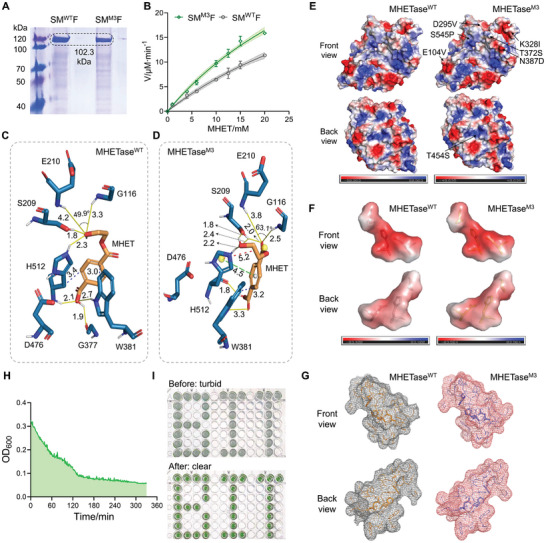
Multiple analysis of SM^WT^F and SM^M3^F. A) SDS‐PAGE and B) enzyme kinetics curves of purified SM^WT^F and SM^M3^F. Molecular docking results of C) MHETase^WT^ and D) MHETase^M3^ with MHET. Yellow solid line: hydrogen bond, blue dashed line: hydrophobic interaction, green dashed line: cation‐π interaction, red dashed line: salt bridge. Surface electrostatic potential distribution of E) the overall protein and F) the catalytic triad. Positive potential regions were shown in blue, neutral regions in white, and negative potential regions in red. Stick models show the Ser‐His‐Asp catalytic triad. G) The solvent accessible surface area of residues within the 5Å around the catalytic triad; H) Variation trend of OD_600_ during MHET turbid solution degradation with SM^M3^F, and I) its before‐and‐after visualization. Values and error bars reflect the mean ± SD of three biological replicates (*n* = 3).

The SM^M3^F mutant exhibited the highest hydrolysis activity toward MHET among these mutants generated from random and rational modifications, so we focused on SM^M3^F for further studies. The mutation residues of SM^M3^F did not noticeably affect the soluble expression of MHETase (**Figure** **6**A). The enzymatic kinetics curves were fitted by the Michaelis–Menten equation. The kinetic parameters of SM^WT^F and SM^M3^F regarding MHET hydrolysis at 37 °C and pH 8.0 were presented in **Table** **2**. The SM^M3^F mutant exhibited a 1.29‐fold increase in *V*_max_, a 1.52‐fold improvement in *k*_cat_/*K*_m_, and a 1.18‐fold reduction in *K*_m_ compared to SM^WT^F (Figure 6B, Table 2), highlighting the effectiveness of the EG‐induced biosensor HTS platform. This comparison of parameters indicated that MHETase^M3^ in SM^M3^F had a stronger affinity toward MHET and showed enhanced catalytic efficiency and turnover rate.

**Table 2:** Kinetic parameters of SM^WT^F and SM^M3^F.

	*V*_max_ [µM min^−1^]	*K*_m_ [mM]	*V*_m_/*K*_m_ [min^−1^]	*k*_cat_ [min^−1^]	*k*_cat_/*K*_m_ [mM^−1^ min^−1^]
SM^WT^F	40.83	51.85	0.0007874	4.083	0.07874
SM^M3^F	52.48	43.92	0.001195	5.248	0.1195

MHETase is an esterase adopting the α/β hydrolase fold, with a substrate‐specific lid domain over the active pocket.^[^ ^23^, ^68^ ^]^ The typical catalytic triad, nucleophilic residue (Ser)‐base (His)‐deprotonated acid (Asp), was located at positions Ser209, His512, and Asp476 in this study.^[^ ^68^ ^]^ To gain insights into the activity enhancement in SM^M3^F, the structure of MHET^M3^ was modeled using SWISS‐MODEL with MHETase^WT^ (PDB ID: 6QZ3) as the model. First, the molecular docking simulations of MHETase^WT^ and MHETase^M3^ with MHET were performed. The binding energies were −2.94 and −3.09 kcal mol^−1^, respectively, revealing a more favorable binding affinity between MHET and MHETase^M3^. Docking results revealed substantial changes in the molecular orientation of MHET within the two complexes. The binding pose of MHET in MHETase^M3^ formed more hydrogen bonds and showed closer distances to Ser209 and His512 residues. The oxyanion hole, formed by the NH groups of Gly116 and Glu210 residues, stabilized the negatively charged oxygen atom in the tetrahedral transition state.^[^ ^23^, ^68^ ^]^ In the MHET‐MHETase^M3^complex, the hydrogen bond distances between MHET and the NH groups decreased, with the angle between the two hydrogen bonds increasing from 49.9° to 63.1°. In addition to the hydrophobic interactions, MHET formed a cation‐π interaction and a salt bridge with the His512 residue in MHETase^M3^ (Figure 6,D). These strengthened and newly formed enzyme‐substrate interactions facilitated the catalytic conformational positioning of MHET in the active pocket, stabilizing the enzyme‐substrate tetrahedral intermediate and ultimately improving catalytic efficiency.

The eight mutations of MHETase^M3^ were distributed around the surface‐exposed loop regions. These distal residues affected the substrate binding and product release in the active pocket through altering the conformational states, and influenced enzyme function indirectly through effects on protein structural stability, conformational changes, and long‐range electrostatic effects. Structural analysis was conducted at both overall and local levels. The overall surface electrostatic potential of MHETase^WT^ ranged from −50.963 (red) to 50.963 (blue), while that of MHETase^M3^ ranged from −48.635 (red) to 48.635 (blue) (Figure 6E). The reduced overall electrostatic potential contributed to maintaining protein conformational stability and lowering the energy barrier for conformational changes. The absolute values of negative surface electrostatic potential for the catalytic triad of MHETase^WT^ and MHETase^M3^ were 83.400 and 83.964, respectively, with an increase of 0.564 (Figure 6F). The catalytic mechanism of MHETase involved acylation and deacylation, both being triggered by the protonation of the nitrogen atom of His512 residue, with the electronegative oxygen atom acting as a nucleophile to attack the carbonyl C (electropositive center) (Figure S7, Supporting Information). The enhanced electron cloud density around the catalytic triad in MHETase^M3^ facilitated deprotonation, enhancing the nucleophilic reactivity of the attacking oxygen atom, and stabilizing the tetrahedral transition state to lower the reaction activation energy. Docking analysis also demonstrated a reduced binding energy of MHETase^M3^, supporting this inference. Additionally, the solvent‐accessible surface areas within 5 Å of residues around the catalytic triad of MHETase^WT^ and MHETase^M3^ were increased from 2217.481 to 2487.920 Å^2^, facilitating substrate binding and product release (Figure 6G). Overall, these mutations of MHETase ^M3^ affected MHET conversion beneficially and synergistically across multiple aspects. In future studies, we will proceed with the computational enzyme design of MHETase based on its conformational dynamics, and generate smart libraries by predicting distal mutation hotspots through reconstructing the enzyme conformational landscape coupled with correlation‐based tools (e.g. the shortest path map^[^ ^71^ ^]^), and leverage our EG‐induced biosensor‐based HTS tool to identify superior variants.

To visually show the degradation efficiency of SM^M3^F, purified SM^M3^F was added to a turbid solution containing 25 mg mL^−1^ of MHET in 50 mM Tris‐HCl (pH 8.0) to a final concentration of 250 µM, and shaken at 37 °C. The degradation of the poorly soluble MHET in water increased the clarity of the initially turbid solution, reflected by a steady decrease in OD_600_ values (Figure 6H). Figure 6I clearly shows the before‐and‐after comparison, providing a straightforward visual demonstration.

### 2.8 A Chemical‐Enzymatic ‘PET‐BHET‐MHET‐TPA‐PCA‐GA’ Upcycling Route

**Figure 7:**
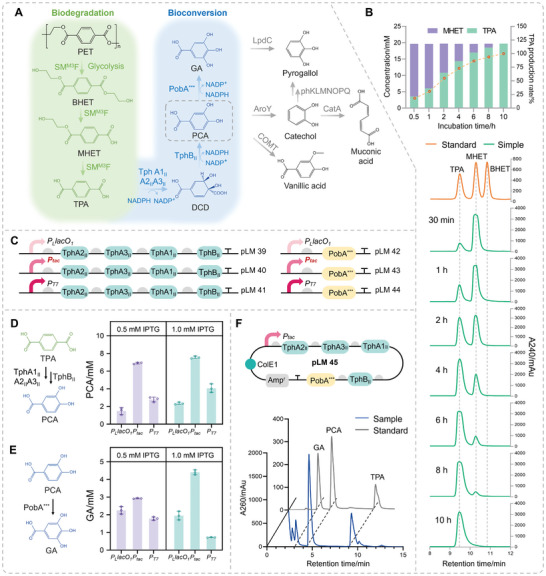
Proof of concept of the PET waste upcycling route. A) Metabolic pathways for intracellular TPA internalization. Proteins not described in the main text with UniProt IDs were as follows. LpdC: F9US27, gallate decarboxylase. AroY: B2DCZ6, 3,4‐dihydroxybenzoate decarboxylase. COMT: Q6T1F5, caffeic acid 3‐O‐methyltransferase. PhKLMNOPQ: Q93JV2, Q93JV2, Q84AQ3, Q84AQ2, Q84AQ1, Q84AQ0, A0A0S2UPA7, phenol hydroxylase. CatA: Q6XUN7, catechol 1,2‐dioxygenase. B) UHPLC detection during BHET degradation with the SM^M3^F crude enzyme extract. C) TPA transformation pathways and PCA transformation pathways. D) Optimization of TPA conversion to PCA. E) Optimization of PCA conversion to GA. F) Plasmid design of TPA to GA, and UHPLC detection of degradation products. Values and error bars reflect the mean ± SD of three biological replicates (*n* = 3).

The circular plastic bioeconomy has emerged as a vivid field aiming at reducing and upcycling plastic waste to promote sustainable production and consumption. In this study, we integrated a degradation pathway with a biosynthesis pathway to upcycle PET waste into valuable products, specifically protocatechuic acid (PCA) and gallic acid (GA), as illustrated in **Figure** **7**A. PET was first degraded into BHET, which was then efficiently broken down into TPA and MHET. Subsequently, TPA was converted into 1,2‐dihydroxy‐3,5‐cyclohexadiene‐1,4‐dicarboxylate (DCD) by TPA 1,2‐dioxygenase (TPADO), composed of an oxygenase component (TphA2_II_A3_II_) and a reductase component (TphA1_II_). DCD was then catalyzed into PCA by DCD dehydrogenase (TphB_II_). PCA, a phenolic acid widely used in pharmaceuticals, functional foods, cosmetics, and materials industry,^[^ ^72^ ^]^ was further transformed into GA by p‐hydroxybenzoate hydroxylase (PobA^***^).^[^ ^73^ ^]^ GA is noted for its antioxidant, antibacterial, antitumor, and antiviral properties, and serves as an important raw material in pharmaceuticals and food additives.^[^ ^73^ ^]^

We hold the view that the key challenge in PET degradation lies not just in breaking down the PET structure, but in obtaining a single depolymerization product of high purity. High‐crystallinity PET can be efficiently degraded by chemical glycolysis within 3 h, primarily yielding BHET, with smaller amounts of MHET and PET oligomers.^[^ ^74^ ^]^ Mesophilic FastPETase efficiently depolymerizes low‐crystallinity PET within 24 h at mild temperature, yielding BHET and MHET.^[^ ^20^ ^]^ For post‐consumer PET with complex crystalline structures, a chemical‐enzymatic strategy is broadly applicable for industrial PET treatment. Therefore, BHET, a “secondary‐level” substrate on the “PET‐BHET‐MHET‐TPA” degradation chain, was selected as the starting substrate for further high value‐added bioconversion study. The SM^M3^F crude enzyme extract exhibited remarkable power toward BHET, functioning as an excellent BHETase. 5 g L^−1^ (19.67 mM) BHET was almost completely degraded within 30 min at 37 °C, producing 3.54 mM TPA and 16.12 mM MHET (Figure 7B). Relieved MHET inhibition by SM^M3^F chimera promoted BHET degradation, whose degradation efficiency using the SM^M3^F biocatalyst was 1108‐fold faster than *Pseudomonas aeruginosa* PR3 strain (5 g L^−1^ BHET),^[^ ^75^ ^]^ 144‐fold faster than *Rhodococcus biphenylivorans* GA1 and *Burkholderia* sp. EG1 microbial consortia (10 mM BHET),^[^ ^76^ ^]^ 18‐fold faster than ΔChryBHETase (5 mM BHET),^[^ ^77^ ^]^ 6‐fold faster than ΔBsEst (5 mM BHET).^[^ ^77^ ^]^ The TPA production rate (*P_TPA_*) increased over time and was modeled by nonlinear fitting, *P_TPA_* = 28.303ln(*t*)  + 34.76 (R^2^ = 0.9957), where *t* represented time (h). The BHET complete degradation half‐life was ≈1.7 h. After 10 h of incubation, the released MHET was 100% converted into TPA and EG (Figure 7B), with an initial enzyme activity of 0.0478 U mL^−1^ toward MHET. This result demonstrated the notable practicality of SM^M3^F in recovering homogeneous TPA, overcoming challenges of downstream applications involving PET repolymerization and high‐value chemical synthesis that were contaminated by BHET and MHET.

To optimize GA production, we fine‐tuned the pathways from TPA to PCA and from PCA to GA, respectively (Figure 7C). The overexpression of pathways was regulated by *P_L_lacO_1_*, *P_tac_*, or *P_T7_* promoters and induced with 0.5 or 1.0 mM isopropyl β‐D‐1thiogalactopyranoside (IPTG). UHPLC analysis revealed that the highest yields were achieved with a medium transcription rate using *P_tac_* and induced with 1.0 mM IPTG (Figure 7,E). Therefore, we designed plasmid pLM45 to co‐express TphA2_II_A3_II_A1_II_, TphB_II_, and PobA^***^ under a single *P_tac_* promoter (Figure 7F). A sequential one‐pot strategy was adopted using the SM^M3^F crude enzyme (in 50 mM Tris‐HCl, pH8.0) and B‐LM45 whole‐cell catalysts to produce GA from BHET. This approach eliminates the need for complex and costly purification steps, highlighting the potential advantages of scaling up production to an industrial level. The BHET post‐degradation solution without downstream purification was supplemented with 10 × M9Y medium and incubated with the GA biosynthetic strain, B‐LM45. After 24 h of cultivation, 997.16 mg L^−1^ (6.47 mm) PCA and 411.69 mg L^−1^ (2.42 mM) GA were detected, with a TPA utilization rate of 45.2% (Figure 7F). Bacteria capable of growing on TPA as a sole carbon source are highly expected to construct transformation pathways for PET upcycling. We proposed a chemo‐biological process with the effective SM^M3^F biocatalyst for recycling PET waste of arbitrary crystallinity, and provided more insights into low‐energy consumption industrial practices through an open‐loop upcycling route: ‘PET‐BHET‐MHET‐TPA‐PCA‐GA’.

## 3 Conclusion

The advancement of recycling and upcycling platforms leveraging PET degrading microbes and enzymes has become a top priority. We developed an advanced fluorescent HTS technique utilizing the first EG‐induced BmoR‐based biosensor to directly detect EG production, a PET degradation end‐product. This “degrading‐biosensing” two‐step HTS protocol is applicable for evolving both mesophilic and thermophilic PET degrading enzymes, with the only difference being the substrate incubation temperatures. Bio‐based polyesters of EG with TPA structure analogs (e.g., thiophen‐, furan‐ or pyridine‐dicarboxylic acid) are promising PET substitutes, such as polyethylene‐2,5‐furandicarboxylate (PEF).^[^ ^78^, ^79^ ^]^ This work extends existing HTS tools to target hydrolases for EG‐based plastic polymers. Additionally, we anticipate the EG‐responsive HTS tool will be instrumental in evolving PETase into ‘PEXase’ and MHETase into ‘MHEXase’ for completely degrading emerging bio‐polyesters (PEX or MHEX). Moreover, this biosensor can serve as a versatile tool in various EG‐related biological and industrial applications, such as detecting EG pollution in wastewater; regulating EG‐associated metabolic networks, and identifying EG or EG‐derived overproducers. The highly EG‐responsive BmoR mutants, BmoR^N207S^ and BmoR^F177L/Q285L^, were generated by FACS‐assisted TF randomly modification, improving the sensitivity, dynamic range, and operation range of biosensors. Combining experimental and computational results, we analyzed how specific mutations influenced the sensing performance of BmoR. It is also worth mentioning that the promiscuous ligand spectrum of BmoR holds promise for engineering it into other dihydric alcohol‐responsive TFs, offering potential HTS tools for multiple polyester hydrolases.

To address the issue of low or non‐expression of heterologous proteins in *E. coli*, we proposed a novel strategy of adding double‐terminal solubilization proteins, considerably promoting the functional soluble expression of MHETase in the form of SMF fusion proteins. The strategy of ‘leveraging highly soluble proteins to drive the soluble expression of poorly soluble proteins’ presents an approach for the functional expression of proteins with poor solubility. Future efforts also should focus on increasing enzyme soluble expression, a necessary step toward facilitating large‐scale applications and broader adoption. Utilizing the biosensor‐based HTS tool, three SMF chimera mutants with increased activity were generated after one round of protein engineering. The comprehensive analysis of SM^M3^F chimera through sequence and structural analysis, mutation site analysis, enzymatic assays, computational analysis, and mechanistic explanations, provided deeper insights into the structure‐function relationships of MHETase for further engineering efforts. Despite the achievements with our fluorescence‐based HTS system, there is still potential for further optimization. Free‐diffusing EG molecules can cause cross‐talk among individual degradation variant cells, hindering the coupling of the BmoR‐based biosensor with FACS for identifying truly highly fluorescent variants. The fluorescence‐activated droplet sorting (FADS) technique, combined with microfluidic devices that encapsulate cells in droplets, offers a promising solution to prevent EG molecules from diffusing evenly across cells by generating physical barriers. In our “degrading‐biosensing” two‐step protocol, degradation variant cells are in encapsulated in microdroplets, and sensing cells harboring biosensor plasmids (*E. coli* X‐EG3/EG7) are pico‐injected into each droplet after a period of enzyme induction and EG production. High‐fluorescence droplets exhibiting high hydrolytic activity are then sorted using the FADS‐assisted ultra‐HTS system, facilitating large‐scale library screening for EG‐based plastic hydrolases (Figure S8, Supporting Information).

Glycolysis pretreatment overcomes current limitations in degrading high‐crystallinity PET by PETase, and the SM^M3^F biocatalyst efficiently suppresses MHET inhibition. We successfully proved the concept of the ‘PET‐BHET‐MHET‐TPA‐PCA‐GA’ upcycling route with a chemical‐enzymatic hydrolysis process, showcasing practicality and feasibility in addressing plastic pollution. In summary, our work offers a visual HTS tool employing an EG‐induced BmoR‐based biosensor for advancing the directed evolution of enzymes involved in PET degradation and proposes a complete chemical‐enzymatic degradation route, facilitating the DBTL cycle of EG‐based plastic degrading enzymes and advancing the industrialization process of enzymatic plastic waste recycling and plastic‐based circular bioeconomy. This study points toward biosensor‐coupled recycling and remediation strategies to answer the current and future plastic pollution imposed on ecosystems and human health.

## 4 Experimental Section

### Chemicals, reagents, and kits

Chemicals, reagents, and kits were shown in Supporting Information.

### Strains and Culture Conditions

*E. coli* XL10‐Gold was used for constructing plasmids and BmoR mutation library, and biosensing. *E. coli* BL21(DE3) was employed for protein expression, MHETase mutation library construction, biodegradation, and bioconversion. Luria–Bertani (LB) medium contained 10 g L^−1^ tryptone, 5 g L^−1^ yeast extract, 10 g L^−1^ NaCl. Solid medium was prepared with 20 g L^−1^ agar. Modified M9 (M9Y) medium contained 6.78 g L^−1^ Na_2_HPO_4_, 3 g L^−1^ KH_2_PO_4_, 0.5 g L^−1^ NaCl, 1 g L^−1^ NH_4_Cl, 1 mM MgSO_4_, 0.1 mM CaCl_2_, 10 mg L^−1^ vitamin B1, 40 g L^−1^ glucose and 4 g L^−1^ yeast extract. Antibiotic concentrations were 100 µg/mL ampicillin, and 50 µg mL^−1^ kanamycin (Kan_50_). Strains were listed in Table S2 (Supporting Information).

### Gene synthesis and Plasmid Construction

All plasmids, primers, codon‐optimized coding sequences, and PCR details were listed in Tables S3, S4, S5, and S6 (Supporting Information), respectively. All plasmids were constructed through Gibson Assembly^[^ ^80^ ^]^ and sequenced at GENEWIZ (Tianjin, China). Plasmid pYH1^[^ ^35^ ^]^ was the original plasmid to construct biosensing plasmids. Vector pET28a(+) was the backbone to construct biodegradation plasmids.

### Random Library Construction and Site‐Directed Mutagenesis

Error‐prone PCR (ep‐PCR) was employed to generate the random BmoR and MHETase libraries with varying mutation rates, as detailed in Supporting Information, Table S6 (Supporting Information). For conducting site‐directed mutagenesis, a pair of primers with partially reversed homologous overlapping regions (10–15 bp) were designed. The resulting linearized PCR fragments were digested by *DpnI* restriction endonuclease (Beijing, China) and then directly transformed.

### Dose‐Response Testing of the BmoR‐Based Biosensor

To evaluate the sensing performance of BmoR‐based biosensors, dose‐responses were conducted in 96‐DWPs and plotted using GraphPad Prism 8.0.2 software, as described in Supporting Information. All fluorescence measurements were normalized to OD_600_ values, i.e., normalized fluorescence intensity (NFI) = sample fluorescence value/OD_600_ (a.u., arbitrary units).

### FACS‐Based BmoR Mutation Library Primary Screening

The BmoR random mutation library was induced at a final concentration of 10 mM EG at 37 °C overnight, then was diluted into phosphate saline buffer for three sequential FACS analysis, using a BD FACS AriaII flow cytometer (BD Biosciences) as described in Supporting Information.

### Protein Structure Prediction, Molecular Docking, and in Silico Analysis

Structures of BmoR^WT^ monomer, BmoR^N207S^ monomer, BmoR^F177L/Q285L^ monomer, and SMF chimera were predicted via AlphaFold 2.^[^ ^62^ ^]^ The BmoR^WT^ hexamer and BmoR^F177L/Q285L^ hexamer were modeled via AlphaFold 3.^[^ ^63^ ^]^ The structure of MHETase^M3^ was modeled in SWISS‐MODEL^[^ ^81^ ^]^ with the model of 6QZ3 (PDB ID). Structures of ligands (EG, MHET) were downloaded from PubChem. Semi‐flexible molecular docking simulations were performed using AutoDock 4.2 as described in Supporting Information. The interchain interaction, surface electrostatic potential distribution, and solvent‐accessible surface area were analyzed by PyMOL (version 2.4).

### Protein Expression, Purification, and Detection

*E. coli* BL21(DE3)‐based protein expression, Ni‐resin affinity chromatographic purification, SDS‐PAGE, and Bradford protein assay were described in Supporting Information.

### UHPLC Analysis

The qualitative and quantitative analysis of BHET, MHET, TPA, PCA, and GA was performed using UHPLC, as described in Supporting Information.

### MHET Initial Concentration Optimization

The 2 mL of B‐LM11 seed culture was inoculated into LB/Kan_50_ medium with 10 µL (5 mM), 20 µL (10 mM), and 30 µL (15 mM) of 1 M MHET (in DMSO) and grown at 37 °C, 220 rpm until OD_600_ reached 0.6. SMF protein was induced with 0.2 mM IPTG. After 12 h of induction at 16 °C, cultures were incubated at 37 °C for MHET degradation. Samples were taken at 12, 24, 36, and 48 h and analyzed by UHPLC.

### HTS of MHETase Mutation Library and Evaluation of Site‐Directed Mutation Mutants

The MHETase random mutation library was screened by evaluating the EG production via the biosensing strain, X‐EG7, as described in Supporting Information. The catalytic performance of site‐directed mutants was evaluated in tubes. Overnight seed cultures were inoculated into 2 mL of LB/Kan_50_ medium with 10 mM MHET and grown at 37 °C, 220 rpm until OD_600_ reached 0.6. Induction was performed with 0.2 mM IPTG for 12 h at 16 °C. Cultures were then incubated for 48 h at 37 °C for MHET degradation. TPA production was measured using UHPLC.

### In Vitro Enzyme Activity Assays

SM^WT^F and SM^M3^F purified enzymes (1 µM) were incubated with MHET (1–20 mM) in 50 mM Tris‐HCl (pH 8.0) at 37 °C for 1 h, and reactions were quenched by adding an equal volume of 100% methanol and heating at 85 °C for 10 min. The “no enzyme” controls (using buffer instead) were included to evaluate the non‐enzymatic MHET hydrolysis background. The TPA release was measured by UHPLC. The non‐linear curves were fitted by Michaelis–Menten Equation (1) using GraphPad Prism 8.0.2 software to calculate *V*_max_, *K*_m,_ and *k*_cat_ values. The enzyme activity of the crude enzyme extract (1 U mL^−1^) was defined as 1 mL of crude enzyme extract converting 1 µmol substrate to 1 µmol product per minute at 37 °C, pH 8.0. All reactions were performed with three replicates.

(1) $$V=\left(V_{\max}+\left[\mathrm{MHET}\right]\right)/\left(K_{m}+\left[\mathrm{MHET}\right]\right)$$

where *V* is the reaction rate, *V*_max_ is the maximum reaction rate, *K_m_* is the Michaelis constant, [MHET] is the concentration of MHET.

### Bioconversion of PET Degradation End‐Products

PCA/GA production optimization, crude enzyme preparation and BHET degradation monitoring, and GA production with post‐degradation solution were described in Supporting Information.

### Statistical Analysis

GraphPad Prism 8.0.2 software was used for data processing. Bar and line graphs are drawn as the means and SD. All experimental results were repeated at least twice to confirm reproducibility. Enzymatic curves were simulated by the “Michaelis‐Menten equation” in GraphPad Prism 8.0.2.

## Conflict of Interest

The authors declare no conflict of interest.

## Author Contributions

M.L. and Z.C. contributed equally to this work. M.L.: Conceptualization, Formal analysis, Investigation, Methodology, Validation, Writing – original draft. Z.C.: Conceptualization, Methodology, Writing – review & editing, Supervision, Project administration, Funding acquisition. W.Z.: Investigation, Validation. T.W., Formal analysis, Resources. Q.Q.: Writing – review & editing, Supervision, Project administration, Funding acquisition. Y.‐X.H.: Conceptualization, Methodology, Writing – review & editing, Supervision, Project administration, Funding acquisition.

## Supporting information



Supporting Information

## Data Availability

The data that support the findings of this study are available from the corresponding author upon reasonable request.
