# Supplementary material for: Customization of Ethylene Glycol (EG)‐Induced BmoR‐Based Biosensor for the Directed Evolution of PET Degrading Enzymes
Source: Adv Sci (Weinh). 2025 Feb 10;12(13):2413205. doi: 10.1002/advs.202413205 (PMC11967783; doi:10.1002/advs.202413205)
Supplement: Supplementary file 1 — Supporting Information [file ADVS-12-2413205-s001.pdf]

ADVANCED SCIENCE
Open Access

Supporting Information

for *Adv. Sci.*, DOI 10.1002/advs.202413205

Customization of Ethylene Glycol (EG)-Induced BmoR-Based Biosensor for the Directed Evolution of PET Degrading Enzymes

Min Li, Zhenya Chen, Wuyuan Zhang, Tong Wu, Qingsheng Qi and Yi-Xin Huo**

Supporting Information

Customization of ethylene glycol (EG)-induced BmoR-based biosensor for the directed evolution of PET degrading enzymes

M. Li, Z. Chen, W. Zhang, T. Wu, Q. Q, Y.-X. Huo**

**Corresponding authors. Email: chenzhenya@bit.edu.cn (Z.C.); huoyixin @bit.edu.cn (Y.-X.H.)*

Experimental Section

1. Chemicals, reagents, and kits
2. Gene synthesis
3. SacB negative-selection verification
4. Random mutation library construction by error-prone PCR
5. Dose-response testing of BmoR-based biosensors
6. FACS-based BmoR mutation library primary screening
7. Molecular docking simulations
8. Protein expression, purification, and detection
9. UHPLC analysis
10. High-throughput screening of MHETase mutation library
11. Bioconversion of PET degradation end-products

Supplementary Figures

Figure S1. Dose-response curves
Figure S2. Interchain interaction analysis
Figure S3. SDS-PAGE analysis
Figure S4. Location distribution of disulfide bonds
Figure S5. *In silico* analysis of the influence of fusion expression on catalytic mechanisms
Figure S6. The fold change of TPA production of SMF site-directed mutagenesis mutants.
Figure S7. Catalytic mechanism of MHET degradation by MHETase
Figure S8. Ultra-high throughput screening strategy based on the EG-induced BmoR-based biosensor and fluorescence-activated droplet sorting (FADS) platform

Supplementary Tables

Table S1. Error-prone PCR amplification system composition
Table S2. List of strains used in this study
Table S3. List of plasmids used in this study
Table S4. List of oligonucleotides used in this study
Table S5. List of protein coding sequences used in this study
Table S6. PCR and plasmids construction details

Experimental Section

1. Chemicals, reagents, and kits

All chemicals were purchased from Aladdin (Shanghai, China), Macklin (Shanghai, China) and Solarbio Science & Technology Co., Ltd. (Beijing, China). *DpnI* restriction endonuclease was purchased from New England Biolabs (Beijing, China). The DNA gel purification kit and plasmid extraction kit were sourced from TransGen Biotech (Beijing, China). DNA polymerase, ClonExpress MultiS One Step Cloning Kit, and One-Step PAGE Gel Fast Preparation Kit (10%) were purchased from Vazyme Biotech Co., Ltd (Nanjing, China). Oligonucleotides (10 μM) were synthesized by Genewiz (Tianjin, China). DNA/protein marker and DNA/protein loading buffer were obtained from TransGen Biotech (Beijing, China). HisSep Ni-NTA Agarose Resin and the BSA standard were procured from Yeasen Biotechnology Co., Ltd (Shanghai, China).

2. Gene synthesis

Genes encoding MHETase, $TphA1_{II}$, $TphA2_{I}$, $TphA3_{II}$ and $TphB_{II}$ were artificially synthesized by overlap extension PCR using 45−60 nt oligonucleotides with homologous complementary regions. The primer mix was prepared by using 5 μL of each primer. The first-round PCR system contained 5 μL of primer mix, 10 μL of 2 × Phanta Flash Master Mix (Vazyme, Nanjing, China) and 5 μL of ddH_2O. The second-round PCR system contained 3 μL of the first-round PCR product, 1 μL of the first primer, 1 μL of the last primer, 25 μL of 2 × Phanta Flash Master Mix (Vazyme, Nanjing, China) and 20 μL of ddH_2O. The PCR reaction procedure was similar to conventional PCR. The PCR products were then confirmed and purified by agarose gel electrophoresis.

3. SacB negative-selection verification

After overnight growth on an LB/Amp_{100} agar plate, a single colony of the X-EG2 strain was picked and inoculated into 4 mL of LB/Amp_{100} medium at 37 °C and 220 rpm overnight. The seed culture was then subcultured at a 100-fold dilution into LB/Amp_{100} medium supplemented with or without 100 g/L sucrose, respectively, both in the presence of 10 mM isobutanol. The lethal effect of SacB expression induced by isobutanol was evaluated by measuring the growth curves under two conditions.

4. Random mutation library construction by error-prone PCR

Error-prone PCR (ep-PCR) increases the DNA mismatch possibility during amplification. Mutation frequencies were divided based on the number of mutated bases: low (0−4.5

mutations/kb), medium (4.5−9 mutations/kb), and high (9−16 mutations/kb). In this study, these frequencies were achieved by adjusting the concentration of $MnCl_2$ in the PCR reaction mixture. The composition of the ep-PCR system was shown in Table S1. The 100 μL of 10 × unbalanced dNTPs contained 2 μL of dATP (100 mM), 2 μL of dGTP (100 mM), 8 μL of dCTP (100 mM) and 8 μL of dTTP (100 mM), supplemented with 80 μL of ddH_2O. The reaction procedure was as follows: 30 cycles of 94 °C denaturation for 30 s, 56 °C annealing for 1 min/kb and 72 °C extension for 30 s. Hieff® Taq DNA Polymerase was purchased from Yeasen Biotechnology Co., Ltd (Shanghai, China). The ep-PCR products were confirmed and purified through agarose gel electrophoresis.

Table S1. Error-prone PCR amplification system composition

Components	Volume/μL	Final concentration
10 × unbalanced dNTPs	10	—
Tris (100 mM, pH 8.3)	10	10 mM
KCl (500 mM)	10	50 mM
$MgCl_2$ (100 mM)	4	4 mM
$MnCl_2$ (10 mM)	1.25/2.5/5	0.125/0.25/0.5 mM
Plasmid template (10 ng/μL)	1	0.1 ng/μL
Forward primer (10 μM)	2	0.2 μM
Reverse primer (10 μM)	2	0.2 μM
Hieff® Taq DNA Polymerase (5U/μL)	1	0.05 U/μL
ddH_2O	Up to 100	—

BmoR mutation library: Gene sequences encoding the N-terminal and the whole open reading frame of BmoR were amplified using primer pairs BmoR1/BmoR4 and BmoR1/BmoR6, respectively. The corresponding primers used to amplify backbones from plasmid pEG2 were BmoR2/BmoR3 and BmoR2/BmoR5, respectively. The purified products were joined by Gibson Assembly and then transformed into *E. coli* XL10-Gold cells. Cells were spread on LB/Amp_{100} agar plates and incubated overnight at 37 °C. All colonies on the plates were scraped off and resuspended in 20 mL of LB/Amp_{100} medium, shaking at 37 °C overnight. The scale of the mutation pool was roughly estimated by counting the number of colonies on the plates, with a final BmoR mutant library capacity of approximately 50,000.

MHETase mutation library: The gene sequence encoding MHETase was codon-optimized and subcloned into the pET28a(+) vector. The MHETase mutation library was generated using the same ep-PCR procedures as described above. The ep-PCR primer pairs used were SMF1/SMF4. The purified products were transformed into *E. coli* BL21(DE3) cells after Gibson Assembly with pLM11 backbone amplified using primer pairs SMF2/SMF3. Cells were spread on LB/Kan_{50} agar plates and incubated overnight at 37 °C. Single colonies were picked and inoculated into the 96-deep-well plate (96-DWP) with 1 mL of LB/Kan_{50} medium.

5. Dose-response testing of BmoR-based biosensors

E. coli XL10 Gold cells harboring biosensor plasmids were grown as seed cultures in LB/Amp_{100} medium at 37 °C and 220 rpm for 8 h. The 10 μL of seed cultures were subcultured into 990 μL of fresh LB/Amp_{100} medium in 96-DWP supplemented with varying concentrations (0, 6.125, 12.5, 25, 37.5, 50, 75, 100, 125, 150, 175, 200 mM) of EG at 37 °C and 220 rpm. After 16 h of EG induction, 50 μL of all samples per well were added to a black microplate, to which 150 μL of 0.9% NaCl buffer was previously added. The OD_{600} and sfGFP fluorescence intensities at $\lambda_{ex/em}$ = 485/510 nm with a fluorescence gain set at 50, were measured using a microplate reader (BioTek, Synergy H1). All fluorescence measurements were normalized to OD_{600} values, i.e., normalized fluorescence intensity (NFI) = fluorescence value/OD_{600} (a.u., arbitrary units). Data were presented as mean ± standard deviation (SD) of three biological replicates (n = 3). The dose-response curve was plotted using GraphPad Prism 8.0.2 software, with EG concentrations on the horizontal axis and NFI on the vertical axis. The dynamic range was calculated as per the following formula, given as dynamic range = NFI_{max}/NFI_{min}, where NFI_{max} and NFI_{min} represent the maximum and minimum NFI, respectively.

6. FACS-based BmoR mutation library primary screening

The BmoR mutation libraries were thawed on ice, inoculated into 4 mL of LB/Amp_{100} medium, and cultivated overnight at 37 °C and 220 rpm. Given that the libraries were stored in 15% (v/v) glycerol, a potential ligand of BmoR, the culture was washed twice with fresh LB medium before induction to avoid false-positive interference during EG-specific BmoR screening. BmoR libraries were inoculated into LB/Amp_{100} medium at a 100-fold dilution and induced in the presence of 10 mM EG at 37 °C and 220 rpm for 12 h. The 1 mL of the induced culture was centrifuged at 4500 rpm for 3 min and washed twice with phosphate saline buffer (PBS, 0.22 μm filtration and autoclaved). The bacterial solution was diluted to OD_{600} = 1.0, and then 10 μL of the diluted culture was added to 2 mL of PBS buffer for FACS analysis. The PBS buffer comprised 8.0 g/L NaCl, 0.2 g/L KCl, 1.44 g/L Na_2HPO_4 and 0.24 g/L KH_2PO_4. The sfGFP

fluorescence distribution was monitored using a BD FACS AriaII flow cytometer (BD Biosciences) with a 488 nm excitation laser and a 530/30 nm bandpass emission filter. A nozzle diameter of 70 μm was selected for sorting. Cells were captured on the following signal channels: Threshold (voltage 500 V), FITC (voltage 550 V), FSC (voltage 600 V), and SSC (voltage 400 V). The number of droplets collected and the sorting range were determined based on the population distribution and the mutation pool scale. Sorted cells were collected into 2 mL of fresh LB medium without antibiotics. Following a 30 min recovery period at 37 °C and 220 rpm ,100 μg/mL ampicillin was supplied. The overnight culture was subcultured at a 100-fold dilution into LB/Amp_{100} medium containing 10 mM EG for the next round of FACS.

7. Molecular docking simulations

Molecular docking simulations were conducted using AutoDock 4.2 with Genetic Algorithm Parameters and Lamarckian GA (4.2). A standard docking procedure was used to generate 10 independent poses, involving a rigid protein and a flexible ligand with identified torsion angles. Default settings were applied for other parameters. EG was docked into the N-terminal LBD of BmoR using the standard semi-flexible docking protocol. Similarly, MHET was docked into the active pocket of MHETase. The protein-ligand interactions were analyzed by PyMOL software (version 2.4).

8. Protein expression, purification, and detection

Expression: Plasmids for protein expression were transformed into *E. coli* BL21(DE3) cells and grown on LB/Kan_{50} agar plates. Seed cultures were inoculated with single colonies and cultured at 37 °C, 220 rpm. Overnight cultures were subcultured at a 100-fold dilution into shake flasks containing LB/Kan_{50} medium. Cells were grown at 37 °C and 220 rpm to an optical density at 600 nm (OD_{600}) of 0.6−0.8, at which point 0.2 mM IPTG was added to induce protein expression. The temperature was then lowered to 16 °C with shaking at 170 rpm for 12−18 h. Cells were harvested by centrifugation at 4 °C, 4,500 rpm for 15 min, and stored at -80 °C until further use.

Purification: Target proteins were purified by immobilized metal-affinity chromatography using Ni-resin. Frozen cells were resuspended in buffer A (10% (v/v) glycerol, 50 mM Tris-HCl, 20 mM imidazole, 500 mM NaCl, at pH 8.0), and then lysed by sonication at 2 s on, 4 s off, 60% amplitude, 300 W for 30 min. Particulate debris was removed by centrifugation at 13,000 rpm for 30 min at 4 °C. The resulting supernatant was filtered through a 0.22 μm membrane and loaded onto a Ni-resin gravity column equilibrated with buffer A. Non-

specifically bound proteins were washed away using a gradient of buffer A and buffer B (10% (v/v) glycerol, 50 mM Tris-HCl, 200 mM imidazole, 500 mM NaCl, at pH 8.0) not exceeding 100 mM imidazole. The target proteins were eluted using buffer B.

Detection: SDS-PAGE was used to analyze and confirm the expression and purification results. Samples were mixed with 6 × protein loading buffer (TransGen, Beijing, China) and denatured by boiling for 10 min. Gels were run in the Tris-Gly buffer (3.02 g/L Tris, 1 g/L SDS, 18.8 g/L Glycine) at 165 V for 50 min, and stained with the Coomassie Brilliant Blue R250 solution. Protein concentrations were assayed by a Bradford protein assay. A standard curve relating protein concentration to A_{595} (absorbance at 595 nm) was constructed using the Quick Start^{TM} Bradford Protein Assay (BIO-RAD). BSA standards with concentrations of 0, 25, 50, 75, 100, 125, 150, 200, 250, and 500 μg/mL were prepared by diluting a 2 mg/mL BSA standard (Yeasen, Shanghai, China). Sample solutions (10 μL) were pipetted into 190 μL of reagent solution and incubated at room temperature for 5 min before measurement with a microplate reader (BioTek, Synergy H1). All samples were analyzed in triplicate. Standard curve fitting was performed using GraphPad Prism 8.0.2 software through linear regression.

9. UHPLC analysis

UHPLC analysis was performed on an Agilent 1290 Infinity II system (Agilent Technologies, USA). A C18 reverse-phase column (Agilent ZORBAX SB-C18, 4.6 × 250 mm, 5 μm) was employed for analyzing and quantifying standards and samples. Samples and solvents were filtered through a 0.22 μm membrane (Jinteng, Tianjin, China), respectively. The mobile phases consisted of methanol (Mreda, Beijing, China) and water containing 0.6% trifluoroacetic acid (Macklin, Shanghai, China). The column was kept at a constant temperature of 30 ℃ at a flow rate of 1 mL/min. The injection volume was 10 μL. The linear gradient elution conditions were as follows: 15% to 40% (v/v) organic for 10 min, 40% to 100% (v/v) organic for 1 min, 100% (v/v) organic for 2 min, 100% to 15% (v/v) organic for 1 min, and 15% (v/v) organic for 1 min. The UV detector was set to monitor at: 240 nm for TPA, MHET and BHET; 260 nm for PCA and GA. Retention times were recorded as follows: between 3 and 4 min for GA, between 4.4 and 6 min for PCA, between 9 and 10 min for TPA, between 10 and 10.5 min for MHET, between 10.5 and 11.5 min for BHET.

10. High-throughput screening of MHETase mutation library

Single colonies were picked from the MHETase random mutation library plates and inoculated into the 96-DWP1 containing 1 mL of LB/Kan_{50} medium per well, and cultured overnight at

37 °C and 250 rpm. Then, 10 μL of seed cultures were inoculated into 950 μL of LB/Kan_{50} medium supplemented with 10 mM MHET in the 96-DWP2 and grown at 37 °C and 250 rpm for 2.5 h. Afterwards, 50 μL of LB/Kan_{50} medium containing 40 mM IPTG (0.2 mM) was added to induce SMF protein expression at 16 °C. After 12 h of induction, the culture was shifted to 37 °C for MHET degradation. After 48 h of bioconversion, 96-DWP2 was centrifuged at 5000 rpm for 15 min. Then, 940 μL of the supernatant was transferred into a new 96-DWP3, and 50 μL of 20 × LB medium and 10 μL of X-EG7 seed culture were added. The 96-DWP3 was incubated at 37 °C for 16 h, after which fluorescence intensity ($\lambda_{ex/em}$ = 485/510 nm) and OD_{600} were measured. For secondary screening, wells with high NFI were selected, and TPA production was measured using UHPLC. Strains with higher TPA production were sequenced.

11. Bioconversion of PET degradation end-products

Pathway optimization: For PCA/GA production optimization, single colonies of *E. coli* BL21(DE3) harboring different bioconversion plasmids (B-LM39, B-LM40, B-LM41, B-LM42, B-LM43, B-LM44) were picked and inoculated into 3 mL of LB/Amp_{100} at 37 °C and 220 rpm overnight. The next day, 30 μL of seed cultures were transferred into 3 mL of M9Y/Amp_{100} medium supplemented with 10 mM TPA or 10 mM PCA and cultivated at 37 °C and 220 rpm. IPTG (0.5, 1.0 mM) was added after 2 h, and the cultures were incubated at 16 °C and 170 rpm. After 4 h of induction, the cultures were continued at 30 °C and 220 rpm for 24 h.

Crude enzyme extract preparation: Expression and induction conditions were as described above. The harvested cells were resuspended in 50 mM Tris-HCl buffer (pH 8) with a volume of 1/20th of the culture volume. The crude enzyme extract was obtained by sonication, and supplemented with 5 g/L of BHET. Samples were taken at 0.5, 1, 2, 4, 6, 8, 10 h, and reactions were quenched by adding an equal volume of 100% methanol and heating at 85 °C for 10 min.

Post-degradation solution conversion: For GA production using BHET degradation end-products as the substrate in a shake flask, a single colony of B-LM45 was cultured in 3 mL of LB/Amp_{100} medium at 37 °C and 220 rpm overnight. The seed culture was transferred at a 100-fold dilution into a shake flask containing the degraded solution supplemented with 10% (v/v) 10 × M9Y/Amp_{100} medium and cultivated at 37 °C and 220 rpm. IPTG was added to a final concentration of 1.0 mM after 2 h. After 4 h of induction at 16 °C, the culture was continued for 24 h at 30 °C and 220 rpm. All samples were filtered through 0.22 μm membrane and analyzed using UHPLC.

Supplementary Figures

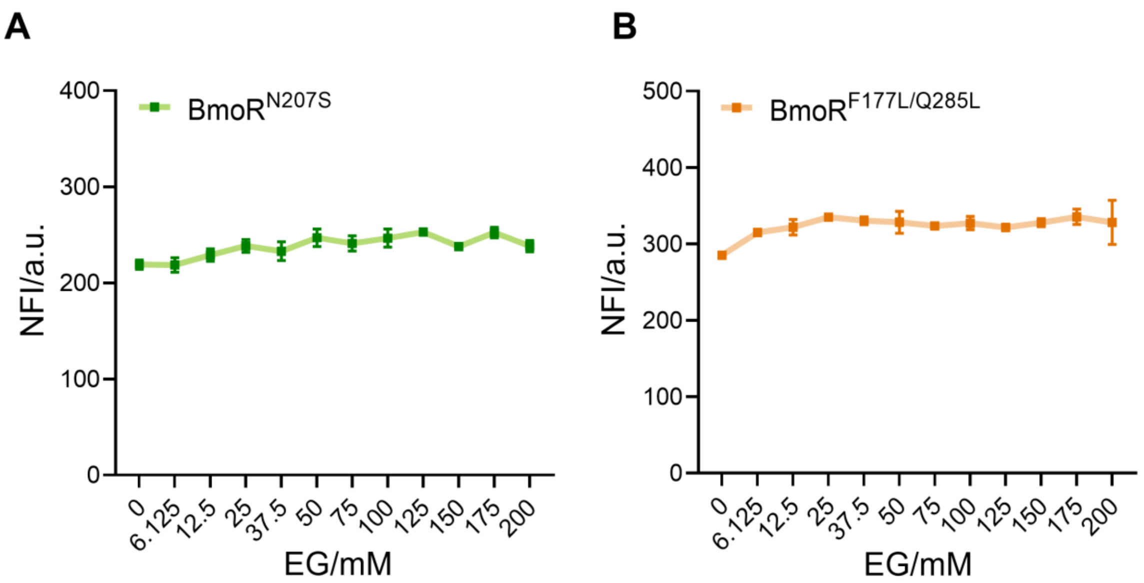

Figure S1. Dose-response curves. Dose-response curves of (A) BmoR^{N207S}-based biosensor (pEG5) and (B) BmoR$^{F177L/Q285L}$-based biosensor (pEG6) with medium-copy origin p15A in the range of 0−200 mM EG. Values and error bars reflect the mean ± SD of three biological replicates (n = 3).

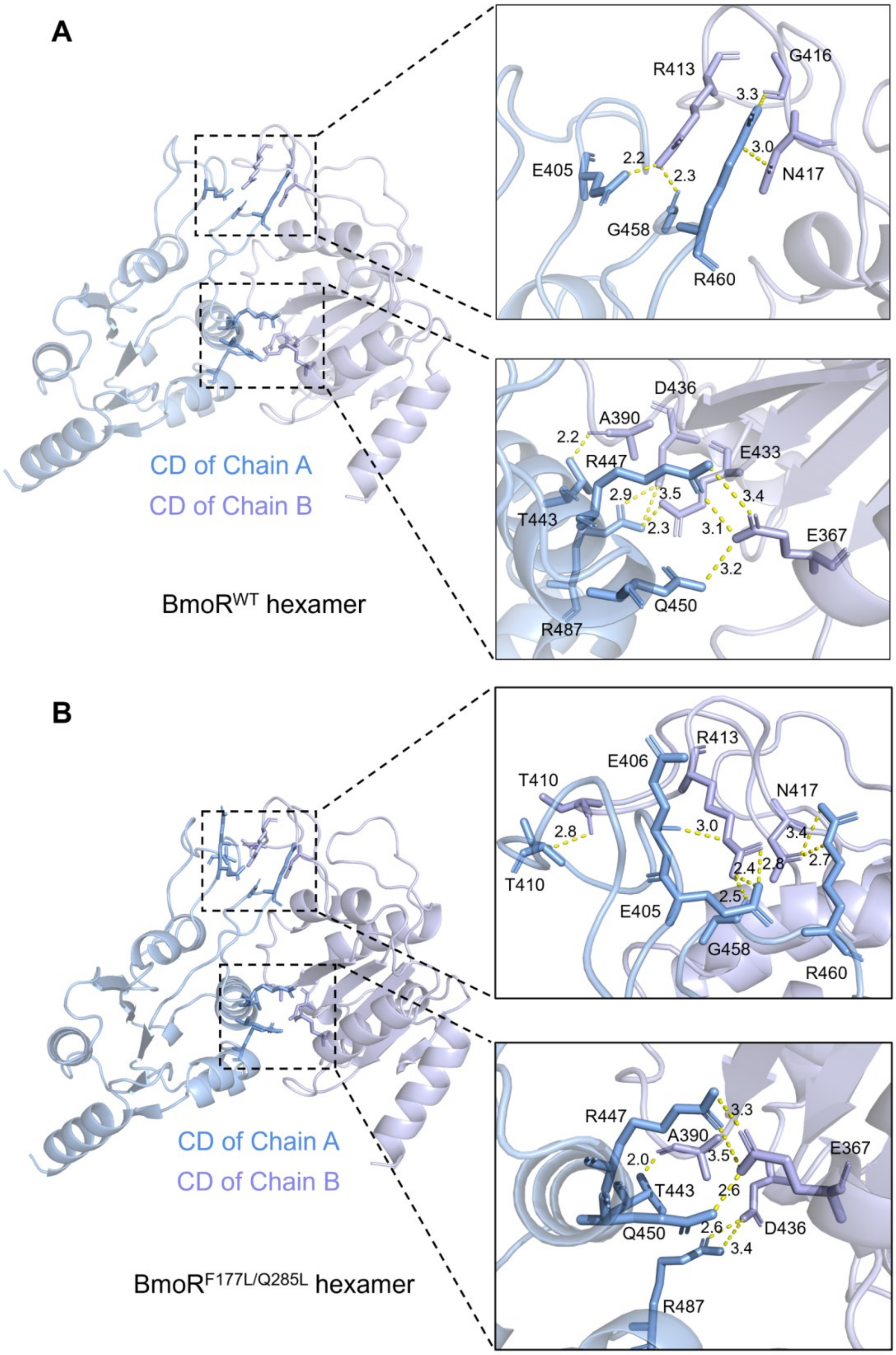

Figure S2. Interchain interaction analysis. Interchain interaction analysis of central domains (CDs) of (A) the BmoR^{WT} hexamer and (B) the BmoR$^{F177L/Q285L}$ hexamer. The CDs were depicted in cartoon representation, with the interacting residues shown as sticks. Yellow dotted line: hydrogen bond.

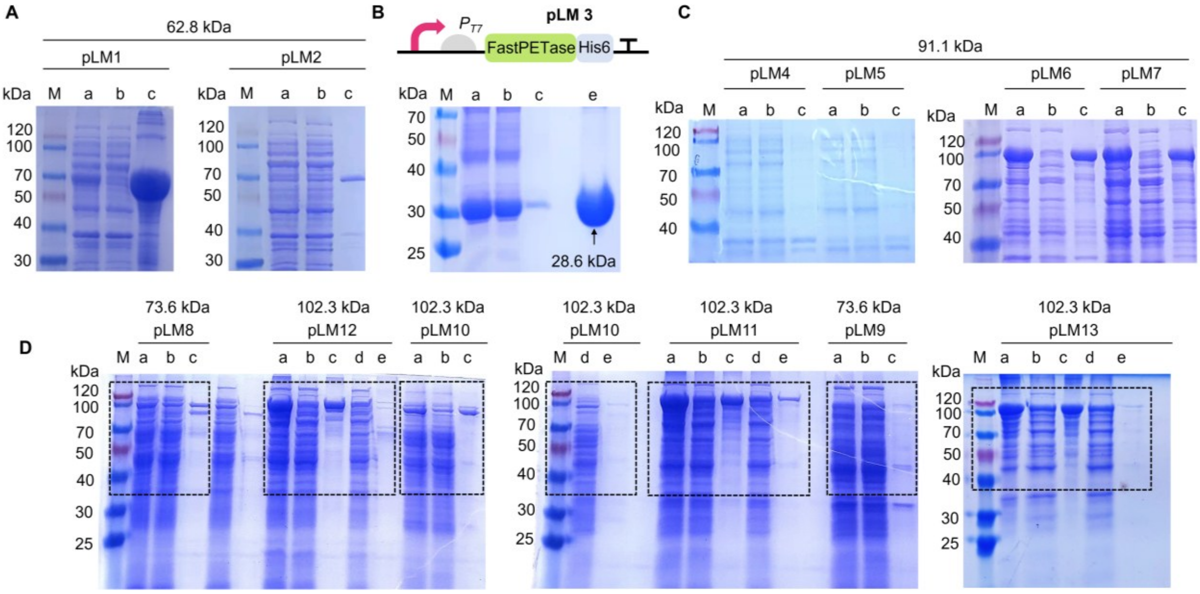

Figure S3. SDS-PAGE analysis. The SDS-PAGE analysis of various plasmids: (A) pLM1 and pLM2, theoretical molecular weight: 62.8 kDa. (B) pLM3, theoretical molecular weight: 28.6 kDa. (C) pLM4, pLM5, pLM6, and pLM7, theoretical molecular weight: 91.1 kDa. (D) pLM8 and pLM9, theoretical molecular weight: 73.6 kDa; pLM10, pLM11, pLM12, and pLM13, theoretical molecular weight: 102.3 kDa. Lane M: marker, Lane a: total protein, Lane b: supernatant, Lane c: sediment, Lane d: flow-through, Lane e: purification.

.

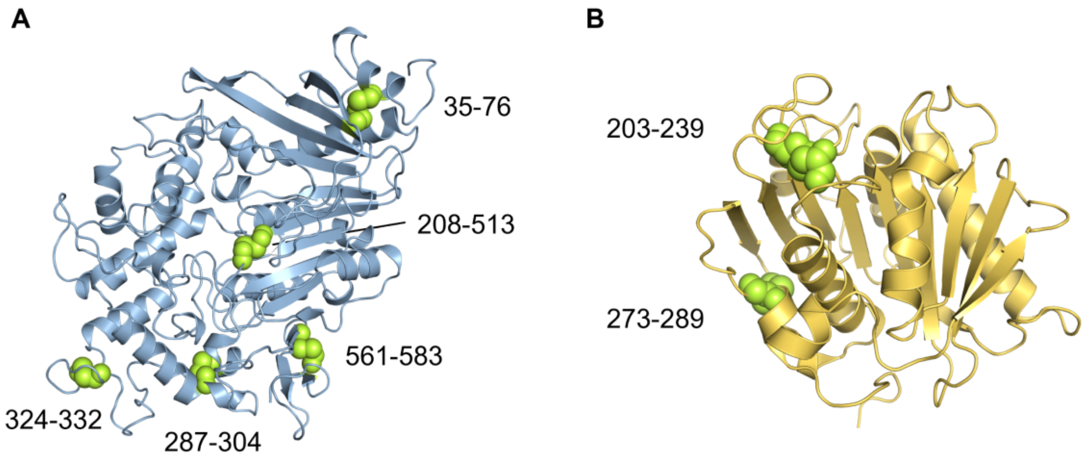

Figure S4. Location distribution of disulfide bonds. Location distribution of disulfide bonds in (A) MHETase (PDB ID: 6QZ3) and (B) FastPETase (PDB ID: 7SH6). The overall structures were represented in cartoon form, with Ser residues shown as spheres. Residues are numbered according to the protein sequences in this study.

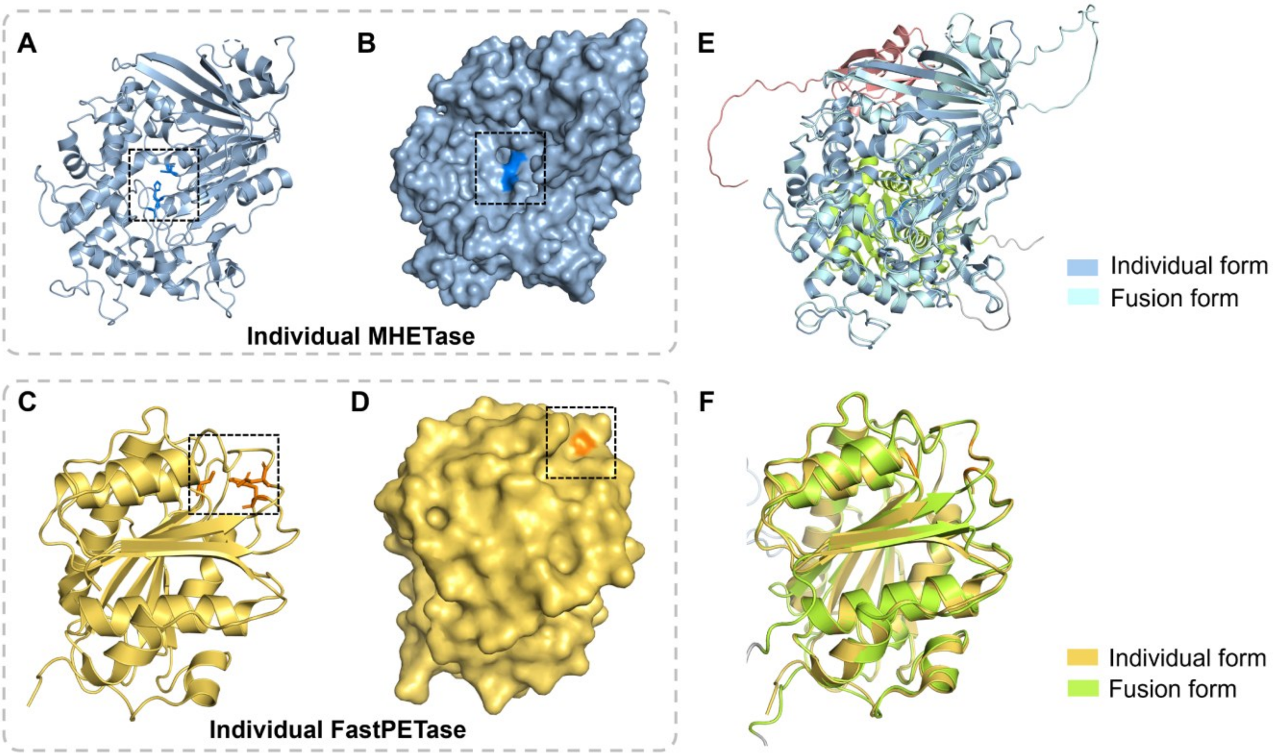

Figure S5. In silico analysis of the influence of fusion expression on catalytic mechanisms. Structures and active pockets (black dotted box) of (A, B) MHETase (PDB ID: 6QZ3) and (C, D) FastPETase (PDB ID: 7SH6). Structure comparison of (E) MHETase and (F) FastPETase in individual and fusion forms.

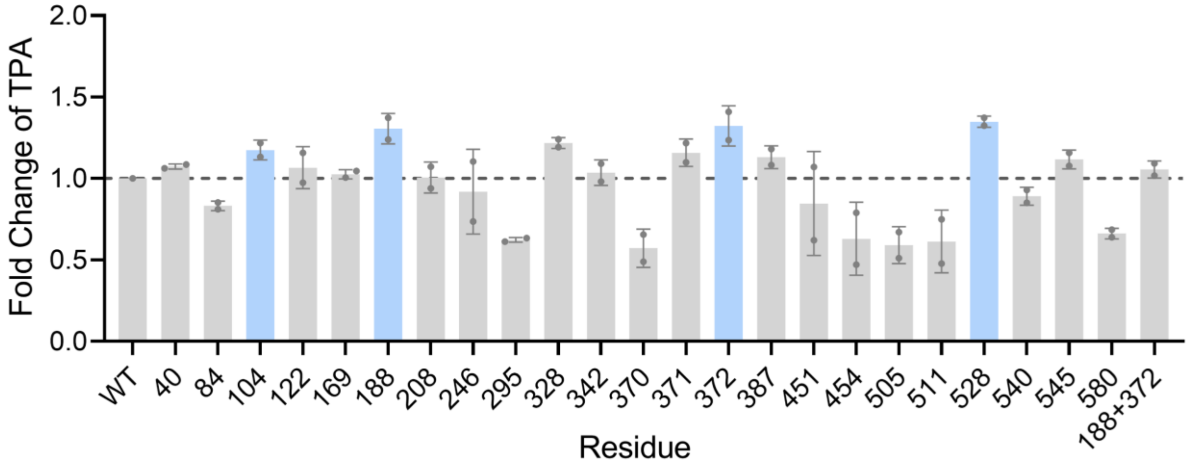

Figure S6. The fold change of TPA production of SMF site-directed mutagenesis mutants. Values and error bars reflect the mean ± SD of two biological replicates (n = 2).

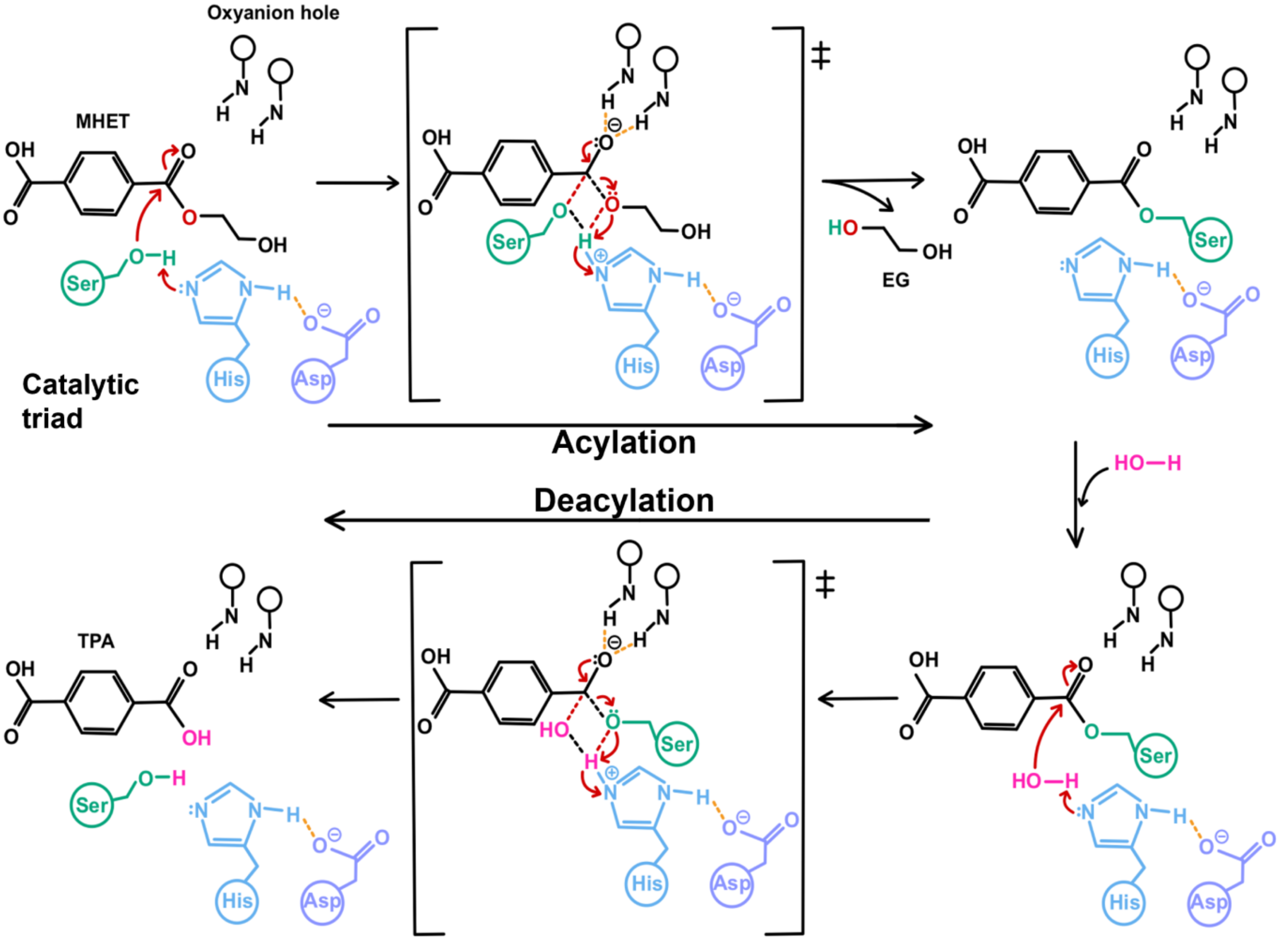

Figure S7. Catalytic mechanism of MHET degradation by MHETase. MHETase possessed a conserved Ser209-His476-Asp512 catalytic triad and an oxyanion hole formed by NH groups from Gly116 and Glu210, adhering to the canonical α/β-serine hydrolase family with reaction mechanism occurring in two stages, acylation and deacylation. The oxyanion hole polarized the ester bond to stabilize the transition state, protecting the negatively charged oxygen atom in the substrate. In the acylation step, the oxygen atom of Ser209 was deprotonated by the nitrogen atom of His512, forming a highly nucleophilic oxygen anion. This initiated a nucleophilic attack on the carboxyl sp2 C atom of MHET, resulting in the formation of an instantaneous acyl-enzyme tetrahedral intermediate. Subsequently, the acylation product EG departed from the active site, with the hydrogen atom originating from Ser209. In the deacylation step, a water molecule approached the nitrogen atom of His512 and was deprotonated. The resulting hydroxide anion initiated a nucleophilic attack on the acyl-enzyme intermediate, facilitating the formation of another transient tetrahedral transition state. Then, the proton transfer from His512 to Ser209 results in the regeneration of the catalytic triad. The product of deacylation was the released TPA[1–4]. Black dashed line: bond about to break. Red dashed line: new bond about to form. Orange dashed line: hydrogen bond. Red arrow: the electron transfer path.

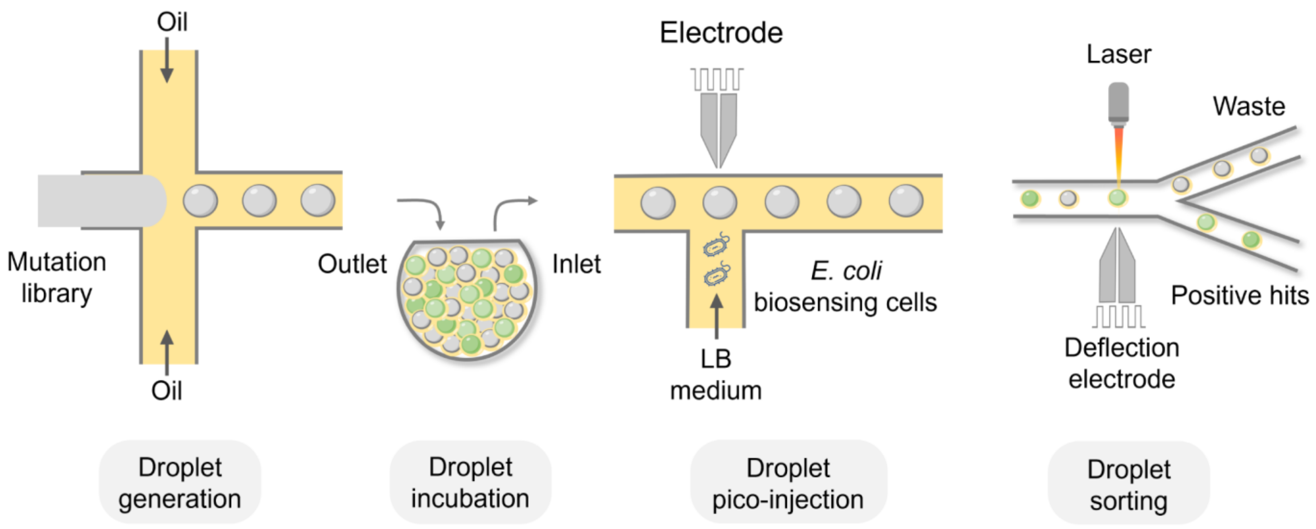

Figure S8. Ultra-high throughput screening strategy based on the EG-induced BmoR-based biosensor and fluorescence-activated droplet sorting (FADS) platform.

Supplementary Tables

Table S2. List of strains used in this study.

Strains	Description	Source
XL10-Gold	*Tet*^{r} Δ(*mcrA*)183 Δ(*mcrCB*-*hsdSMR*-*mrr*)173 *endA1 supE44 thi-1 recA1 gyrA96 relA1 lac Hte* [F′ *proAB lacIqZΔM15* Tn10 (*Tet*^{r}) Amy *Cam*^{r}]	Lab preservation
BL21(DE3)	F^{-}*ompT hsdS (rB^{-}mB^{-}) gal dcm* (DE3)	Lab preservation
X-EG1	XL10-Gold with pEG1	This study
X-EG2	XL10-Gold with pEG2	This study
X-EG3	XL10-Gold with pEG3	This study
X-EG4	XL10-Gold with pEG4	This study
X-EG5	XL10-Gold with pEG5	This study
X-EG6	XL10-Gold with pEG6	This study
X-EG7	XL10-Gold with pEG7	This study
B-LM1	BL21(DE3) with pLM1	This study
B-LM2	BL21(DE3) with pLM2	This study
B-LM3	BL21(DE3) with pLM3	This study
B-LM4	BL21(DE3) with pLM4	This study
B-LM5	BL21(DE3) with pLM5	This study
B-LM6	BL21(DE3) with pLM6	This study
B-LM7	BL21(DE3) with pLM7	This study
B-LM8	BL21(DE3) with pLM8	This study
B-LM9	BL21(DE3) with pLM9	This study
B-LM10	BL21(DE3) with pLM10	This study
B-LM11	BL21(DE3) with pLM11	This study
B-LM12	BL21(DE3) with pLM12	This study
B-LM13	BL21(DE3) with pLM13	This study
B-MHET1	BL21(DE3) with pLM-M1	This study
B-MHET2	BL21(DE3) with pLM-M2	This study
B-MHET3	BL21(DE3) with pLM-M3	This study
B-LM16	BL21(DE3) with pLM16	This study

Strains	Description	Source
B-LM17	BL21(DE3) with pLM17	This study
B-LM18	BL21(DE3) with pLM18	This study
B-LM19	BL21(DE3) with pLM19	This study
B-LM20	BL21(DE3) with pLM20	This study
B-LM21	BL21(DE3) with pLM21	This study
B-LM22	BL21(DE3) with pLM22	This study
B-LM23	BL21(DE3) with pLM23	This study
B-LM24	BL21(DE3) with pLM24	This study
B-LM25	BL21(DE3) with pLM25	This study
B-LM26	BL21(DE3) with pLM26	This study
B-LM27	BL21(DE3) with pLM27	This study
B-LM28	BL21(DE3) with pLM28	This study
B-LM29	BL21(DE3) with pLM29	This study
B-LM30	BL21(DE3) with pLM30	This study
B-LM31	BL21(DE3) with pLM31	This study
B-LM32	BL21(DE3) with pLM32	This study
B-LM33	BL21(DE3) with pLM33	This study
B-LM34	BL21(DE3) with pLM34	This study
B-LM35	BL21(DE3) with pLM35	This study
B-LM36	BL21(DE3) with pLM36	This study
B-LM37	BL21(DE3) with pLM37	This study
B-LM38	BL21(DE3) with pLM38	This study
B-LM39	BL21(DE3) with pLM39	This study
B-LM40	BL21(DE3) with pLM40	This study
B-LM41	BL21(DE3) with pLM41	This study
B-LM42	BL21(DE3) with pLM42	This study
B-LM43	BL21(DE3) with pLM43	This study
B-LM44	BL21(DE3) with pLM44	This study
B-LM45	BL21(DE3) with pLM45	This study

Table S3. List of plasmids used in this study.

Plasmids	Description	Source
pYH1	ColE1; *Amp^r*; *P_{bmoR}-BmoR; P_{bmo}-GFP;*	[5]
pEG1	ColE1; *Amp^r*; *P_{bmoR}-BmoR; P_{bmo}-sfGFP;*	This study
pEG2	ColE1; *Amp^r*; *P_{bmoR}-BmoR; P_{bmo}-sfGFP-SacB;*	This study
pEG3	ColE1; *Amp^r*; *P_{bmoR}-$BmoR^{N207S}$; P_{bmo}-sfGFP-SacB;*	This study
pEG4	ColE1; *Amp^r*; *P_{bmoR}-$BmoR^{F177L/Q285L}$; P_{bmo}-sfGFP-SacB;*	This study
pEG5	p15A; *Amp^r*; *P_{bmoR}-$BmoR^{N207S}$; P_{bmo}-sfGFP-SacB;*	This study
pEG6	p15A; *Amp^r*; *P_{bmoR}-$BmoR^{F177L/Q285L}$; P_{bmo}-sfGFP-SacB;*	This study
pEG7	ColE1; *Kan^r*; *P_{bmoR}-$BmoR^{N207S}$; P_{bmo}-sfGFP-SacB;*	This study
pLM1	ColE; *Kan^r*; *P_{T7}-MHETase-His*;	This study
pLM2	ColE; *Kan^r*; *P_{tac}-MHETase-His*;	This study
pLM3	ColE; *Kan^r*; *P_{T7}-FastPETase-His*;	This study
pLM4	ColE; *Kan^r*; *P_{T7}-MHETase-FastPETase-His*;	This study
pLM5	ColE; *Kan^r*; *P_{tac}-MHETase-FastPETase-His*;	This study
pLM6	ColE; *Kan^r*; *P_{T7}-FastPETase-MHETase-His*;	This study
pLM7	ColE; *Kan^r*; *P_{tac}-FastPETase-MHETase-His*;	This study
pLM8	ColE; *Kan^r*; *P_{T7}-SUMO-MHETase-His*;	This study
pLM9	ColE; *Kan^r*; *P_{tac}-SUMO-MHETase-His*;	This study
pLM10	ColE; *Kan^r*; *P_{T7}-SUMO-MHETase-FastPETase-His*;	This study
pLM11	ColE; *Kan^r*; *P_{tac}-SUMO-MHETase-FastPETase-His*;	This study
pLM12	ColE; *Kan^r*; *P_{T7}-SUMO-FastPETase-MHETase-His*;	This study
pLM13	ColE; *Kan^r*; *P_{tac}-SUMO-FastPETase-MHETase-His*;	This study
pLM-M1	ColE1; *Amp^r*; *P_{lacI}-LacI*; *P_{tac}-SUMO-$MHETase^{M1}$-FastPETase-His*;	This study
pLM-M2	ColE1; *Amp^r*; *P_{lacI}-LacI*; *P_{tac}-SUMO-$MHETase^{M2}$-FastPETase-His*;	This study
pLM-M3	ColE1; *Amp^r*; *P_{lacI}-LacI*; *P_{tac}-SUMO-$MHETase^{M3}$-FastPETase-His*;	This study
pLM16	ColE1; *Kan^r*; *P_{tac}-SUMO-$MHETase^{40}$-FastPETase-His*;	This study
pLM17	ColE1; *Kan^r*; *P_{tac}-SUMO-$MHETase^{84}$-FastPETase-His*;	This study
pLM18	ColE1; *Kan^r*; *P_{tac}-SUMO-$MHETase^{104}$-FastPETase-His*;	This study

Plasmids	Description	Source
pLM19	ColE1; Kan^r; P_{tac}-*SUMO-MHETase*^{122}-*FastPETase-His*;	This study
pLM20	ColE1; Kan^r; P_{tac}-*SUMO-MHETase*^{169}-*FastPETase-His*;	This study
pLM21	ColE1; Kan^r; P_{tac}-*SUMO-MHETase*^{188}-*FastPETase-His*;	This study
pLM22	ColE1; Kan^r; P_{tac}-*SUMO-MHETase*^{208}-*FastPETase-His*;	This study
pLM23	ColE1; Kan^r; P_{tac}-*SUMO-MHETase*^{246}-*FastPETase-His*;	This study
pLM24	ColE1; Kan^r; P_{tac}-*SUMO-MHETase*^{295}-*FastPETase-His*;	This study
pLM25	ColE1; Kan^r; P_{tac}-*SUMO-MHETase*^{328}-*FastPETase-His*;	This study
pLM26	ColE1; Kan^r; P_{tac}-*SUMO-MHETase*^{342}-*FastPETase-His*;	This study
pLM27	ColE1; Kan^r; P_{tac}-*SUMO-MHETase*^{370}-*FastPETase-His*;	This study
pLM28	ColE1; Kan^r; P_{tac}-*SUMO-MHETase*^{371}-*FastPETase-His*;	This study
pLM29	ColE1; Kan^r; P_{tac}-*SUMO-MHETase*^{372}-*FastPETase-His*;	This study
pLM30	ColE1; Kan^r; P_{tac}-*SUMO-MHETase*^{387}-*FastPETase-His*;	This study
pLM31	ColE1; Kan^r; P_{tac}-*SUMO-MHETase*^{451}-*FastPETase-His*;	This study
pLM32	ColE1; Kan^r; P_{tac}-*SUMO-MHETase*^{454}-*FastPETase-His*;	This study
pLM33	ColE1; Kan^r; P_{tac}-*SUMO-MHETase*^{505}-*FastPETase-His*;	This study
pLM34	ColE1; Kan^r; P_{tac}-*SUMO-MHETase*^{511}-*FastPETase-His*;	This study
pLM35	ColE1; Kan^r; P_{tac}-*SUMO-MHETase*^{528}-*FastPETase-His*;	This study
pLM36	ColE1; Kan^r; P_{tac}-*SUMO-MHETase*^{540}-*FastPETase-His*;	This study
pLM37	ColE1; Kan^r; P_{tac}-*SUMO-MHETase*^{545}-*FastPETase-His*;	This study
pLM38	ColE1; Kan^r; P_{tac}-*SUMO-MHETase*^{580}-*FastPETase-His*;	This study
pLM39	ColE1; Amp^r; P_{lacI}-*LacI*; P_L*lacO*$_1$-*TphA2*$_{II}$-*TphA3*$_{II}$-*TphA1*$_{II}$-*TphB*$_{II}$;	This study
pLM40	ColE1; Amp^r; P_{lacI}-*LacI*; P_{tac}-*TphA2*$_{II}$-*TphA3*$_{II}$-*TphA1*$_{II}$-*TphB*$_{II}$;	This study
pLM41	ColE1; Amp^r; P_{lacI}-*LacI*; P_{T7}-*TphA2*$_{II}$-*TphA3*$_{II}$-*TphA1*$_{II}$-*TphB*$_{II}$;	This study
pLM42	ColE1; Amp^r; P_{lacI}-*LacI*; P_L*lacO*$_1$-*PobA****;	This study
pLM43	ColE1; Amp^r; P_{lacI}-*LacI*; P_{tac}-*PobA****;	This study
pLM44	ColE1; Amp^r; P_{lacI}-*LacI*; P_{T7}-*PobA****;	This study
pLM45	ColE1; Amp^r; P_{lacI}-*LacI*; P_{tac}-*TphA2*$_{II}$-*TphA3*$_{II}$-*TphA1*$_{II}$-*TphB*$_{II}$-*PobA****;	This study

Table S4. List of oligonucleotides used in this study.

Primer	Sequence (5′→3′)
BmoR1	aggtgaatgaggagacggtatgt
BmoR2	acataccgtctcctcattcacct
BmoR3	acgctgaatctccgcgtt
BmoR4	aacgcggagattcagcgt
BmoR5	acctaaaggccagcgcc
BmoR6	ggcgctggcctttaggt
lm1	gaacacacaaaggaggaagtgatggttagcaaaggtgaagaactg
lm2	gcggccgctactagtattagctgcctttatacagttcatcc
lm3	taatactagtagcggccgctg
lm4	cacttcctcctttgtgtgttctg
lm7	ggttctggtggtggttctggtggtatgaacatcaaaaagtttgcaaaac
lm8	agcggccgctactagtattatttgttaactgttaattgtccttgttcaag
lm9	cagaaccaccaccagaaccaccgctgcctttatacagttcatcc
lm10	taatactagtagcggccgctg
lm15	accaccagcgcacatggtatatctccttcttaaagttaaac
lm16	cgctgctccgccgcaccaccaccaccaccactga
lm19	tgacaattaatcatcggctcgtataatgggaattgtgagcggataac
lm20	attatacgagccgatgattaattgtcaaatttcgcgggatcgagatc
lm23	cgctaactgctctctggaacaccaccaccaccaccactgag
lm24	acgggttggtctgcatggtatatctccttcttaaagttaaaca
lm26	atgtgcgctggtggtggttc
lm27	agaaccaccaccaccagaaccaccaccacccggcggagcagcgcaagcg
lm28	ttctggtggtggtggttctggtggtggtggttctcagaccaacccgtacgctc
lm30	ttctggtggtggtggttctggtggtggtggttcttgcgctggtggtggttctac
lm31	agaaccaccaccaccagaaccaccaccaccttccagagagcagttagcgg
lm32	cggagcagcgcaagcgaagtta
lm33	gcttgcgctgctccgccgcaccaccaccaccaccactg
lm34	atgcagaccaacccgtac
lm35	taccatgtcggactcagaagtcaatc
lm36	accaccagcgcacattccaccaatctgttctctgtg
lm37	cttctgagtccgacatggtatatctccttcttaaagttaaac

Primer	Sequence (5′→3′)
lm38	cgggttggtctgcattccaccaatctgttctctgtg
lm39	ccaggaaccgtttgagatcgttttggtctgcg
lm40	tttccataggctccgcc
lm41	ggcggagcctatggaaaaaggatctcaagaagatcctttg
lm42	ggatcagaccgattctcgac
lm43	cgatctcaaacggttcctggccttttg
lm44	gtcgagaatcggtctgatccg
lm56	ttagaaaaactcatcgagcatcaaatg
lm57	atgagccatattcaacggg
lm58	catttgatgctcgatgagtttttctaataactgtcagaccaagtttacgag
lm59	cccgttgaatatggctcatactcttcctttttcaatattattgaagc
lm60	actggatgatagattcctgcatcgtacctttctcctctttaatg
lm61	ggtgctcagccggtttaaggatccactagaggcatcaaat
lm64	atgaagactcaagtcgccat
lm65	ttactcgatttcctcgtagggc
lm66	gccctacgaggaaatcgagtaaggatccactagaggcatc
lm67	atggcgacttgagtcttcatcgtacctttctcctctttaatgaattc
lm68	gaattcattaaagaggagaaaggtaccatgaagactcaagtcgcca
lm69	ggtacctttctcctctttaatgaattcttaaaccggctgagcacc
lm70	cctacgaggaaatcgagtaaggatccactagaggcatc
D40A-F	ctctgaaagccggtaacggtgacatggtttg
D40A-R	accgttaccggctttcagagcttcgcaagc
K84R-F	ctatcgctagacgtaccggtatcgacggtt
K84R-R	accggtacgtctagcgatagcaccagaaac
E104V-F	gccggctgtatggaacggtcgtttctt
E104V-R	cgttccatacagccggcatacgcagac
S122T-F	ggttctctgactgctgctaccggttctatc
S122T-R	gtagcagcagtcagagaaccgttggtaccag
D169G-F	ggtctgggcccgcaggctcgtctggac
D169G-R	cctgcgggcccagaccgaaagcaacg
G188S-F	ccaggctagtaaagctgctgttgctcgt
G188S-R	gcagctttactagcctgggtaacctggtc
C208S-F	catcggtagctctgaaggtggtcgtgaag

Primer	Sequence (5′→3′)
C208S-R	accttcagagctaccgatgaagtaagatttg
A246V-F	tctctggtgtttggaccacccagtctc
A246V-R	ggtccaaacaccagagataccagctttc
D295V-F	tctggctgtcggtatcgttgacaactac
D295V-R	acgataccgacagccagaccgtccagag
K328I-F	tggtgctataaccgctgactgcctgtctc
K328I-R	gtcagcggttatagcaccaacgcactgca
K342Q-F	ccgctatccaacgtgctatggctggtccgg
K342Q-R	catagcacgttggatagcggtaacctgaac
S370T-F	ctggtctgactggtaccacctacaaccag
S370T-R	gtggtaccagtcagaccagacataccagc
G371S-F	ggtctgtctagtaccacctacaaccaggg
G371S-R	aggtggtactagacagaccagacatacca
T372S-F	gtctggttccacctacaaccagggttggcg
T372S-R	ggttgtaggtggaaccagacagaccagaca
N387D-F	gggttctttcgactcttctgctaacaacgctc
N387D-R	agcagaagagtcgaaagaacccagccaccaag
H451L-F	ggactggctcggtgctacctctaccgac
H451L-R	gtagcaccgagccagtccatagaagactg
T454S-F	cggtgcttcctctaccgacctggctgct
T454S-R	tcggtagaggaagcaccgtgccagtcca
F505Y-F	tcgtctgtacctggttccgggtatgaacc
F505Y-R	cggaaccaggtacagacgagcgaaaccag
N511D-F	gggtatggaccactgctctggtggtccgg
N511D-R	agagcagtggtccatacccggaaccagga
L528R-F	cccgcgggttgcttgggttgaacgt
L528R-R	caagcaacccgcggggtcagcatgtc
Q540L-F	ccggacctgatctctgcttggtctggt
Q540L-R	gcagagatcaggtccggagcttcacca
S545P-F	tgcttggcctggtaccccgggttacttc
S545P-R	ggggtaccaggccaagcagagatctggtc
N580T-F	cgaagctaccttcgcttgcgctgctccgc
N580T-R	gcaagcgaaggtagcttcggtgttgatgtc

Primer	Sequence (5′→3′)
SMF1	aggctcacagagaacagattggt
SMF2	accaatctgttctctgtgagcct
SMF3	agaccaacccgtacgctcg
SMF4	cgagcgtacgggttggtct
MHET1	atgtgcgctggtggtggttctaccccgctgccgctgccgcagcagcagccgccgcagca
MHET2	aagcagcacgagaagccagcggaaccggcggcggcggcggttcctgctgcggcggctgc
MHET3	ctggcttctcgtgctgcttgcgaagctctgaaagacggtaacggtgacatggtttggcc
MHET4	cgtcacgccaagcagcaacttcaacaacggtagcagcgttcggccaaaccatgtcaccg
MHET5	gctgcttggcgtgacgctgctccggctaccgcttctgctgctgctctgccggaacactg
MHET6	ccgtcgataccggtacgtttagcgatagcaccagaaacttcgcagtgttccggcagagc
MHET7	cgtaccggtatcgacggttacccgtacgaaatcaaattccgtctgcgtatgccggctga
MHET8	ttggtaccagaaccaccttccatgaagaaacgaccgttccattcagccggcatacgcag
MHET9	ggaaggtggttctggtaccaacggttctctgtctgctgctaccggttctatcggtggtg
MHET10	gatggtagcgaagttacgagacagagcagaagcgatctgaccaccaccgatagaaccgg
MHET11	gtctcgtaacttcgctaccatcgctaccgacggtggtcacgacaacgctgttaacgaca
MHET12	tccagaccgaaagcaacggtacccagagcgtccgggttgtcgttaacagcgttgtcgtg
MHET13	ccgttgctttcggtctggacccgcaggctcgtctggacatgggttacaactcttacgac
MHET14	cgagcaacagcagctttaccagcctgggtaacctggtcgtaagagttgtaacccatgtc
MHET15	ggtaaagctgctgttgctcgtttctacggtcgtgctgctgacaaatcttacttcatcgg
MHET16	acagcatcataccttcacgaccaccttcagagcaaccgatgaagtaagatttgtcagca
MHET17	ggtcgtgaaggtatgatgctgtctcagcgtttcccgtctcactacgacggtatcgttgc
MHET18	gagataccagctttcggcagctggtaacccggagcaccagcaacgataccgtcgtagtg
MHET19	ctgccgaaagctggtatctctggtgcttggaccacccagtctctggctccggctgctgt
MHET20	gaaagatttgttgatcagcggaacaccctgagcgtccagaccaacagcagccggagcca
MHET21	gttccgctgatcaacaaatctttctctgacgctgacctgcacctgctgtctcaggctat
MHET22	ccgtcagccagaccgtccagagcgtcgcaggtacccaggatagcctgagacagcaggtg
MHET23	acggtctggctgacggtatcgttgacaactaccgtgcttgccaggctgctttcgacccg
MHET24	cgcactgcagagcctgaccgttagccgggttagcagcggtagccgggtcgaaagcagcc
MHET25	caggctctgcagtgcgttggtgctaaaaccgctgactgcctgtctccggttcaggttac
MHET26	gcagagttaaccggaccagccatagcacgtttgatagcggtaacctgaaccggagacag
MHET27	ctggtccggttaactctgctggtaccccgctgtacaaccgttgggcttgggacgctggt
MHET28	aacgccaaccctggttgtaggtggtaccagacagaccagacataccagcgtcccaagcc
MHET29	ctacaaccagggttggcgttcttggtggctgggttctttcaactcttctgctaacaacg

Primer	Sequence (5′→3′)
MHET30	ccaagaacgagcagagaaaccagaaacacgctgagcgttgttagcagaagagttgaaag
MHET31	ggtttctctgctcgttcttggctggttgacttcgctaccccgccggaaccgatgccgat
MHET32	atgtcgaagtcgaatttcatcatacgagcagcaacctgggtcatcggcatcggttccgg
MHET33	cgtatgatgaaattcgacttcgacatcgacccgctgaaaatctgggctacctctggtca
MHET34	tagaggtagcaccgtgccagtccatagaagactgggtgaactgaccagaggtagcccag
MHET35	ggcacggtgctacctctaccgacctggctgctttccgtgaccgtggtggtaaaatgatc
MHET36	agcagagaaagcagcgtcagacataccgtggtacaggatcattttaccaccacggtcac
MHET37	ctgacgctgctttctctgctctggacaccgctgactactacgaacgtctgggtgctgct
MHET38	ggaaccaggaacagacgagcgaaaccagcagcacccggcatagcagcacccagacgttc
MHET39	ctcgtctgttcctggttccgggtatgaaccactgctctggtggtccgggtaccgaccgt
MHET40	caccacgttcaacccaagcaaccagcggggtcagcatgtcgaaacggtcggtacccgga
MHET41	gcttgggttgaacgtggtgaagctccggaccagatctctgcttggtctggtaccccggg
MHET42	gtacgggcacagcggacgggtacgagcagcaacaccgaagtaacccggggtaccagacc
MHET43	tccgctgtgcccgtacccgcagatcgctcgttacaaaggttctggtgacatcaacaccg
MHET44	cggcggagcagcgcaagcgaagttagcttcggtgttgatgtcaccagaac
FAST1	cagaccaacccgtacgctcgtggtccgaacccgaccgctgcttctctggaagcttctgc
FAST2	cggacgagaaacggtgaaagaacgaacggtgaacggaccagcagaagcttccagagaag
FAST3	tcaccgtttctcgtccgtctggttacggtgctggtaccgtttactacccgaccaacgct
FAST4	ggtgtaacccggaacgatagcgatagcaccaacggtaccaccagcgttggtcgggtagt
FAST5	tcgttccgggttacaccgctcgtcagtcttctatcaaatggtggggtccgcgtctggct
FAST6	agggtagagttggtgtcgatggtgataacaacgaaaccgtgagaagccagacgcggacc
FAST7	cgacaccaactctaccctggaccagccggaatctcgttcttctcagcagatggctgctc
FAST8	tcggagaagaagaggtaccgttcagagaagcaacctgacgcagagcagccatctgctga
FAST9	ggtacctcttcttctccgatctacggtaaagttgacaccgctcgtatgggtgttatggg
FAST10	agcagcagagatcagagaaccaccaccacccatagaccaacccataacacccatacgag
FAST11	gttctctgatctctgctgctaacaacccgtctctgaaagctgctgctccgcaggctccg
FAST12	cagggtcggaacggtaacagaagagaagttggtagaagagtgccacggagcctgcggag
FAST13	ttaccgttccgaccctgatcttcgcttgcgaaaacgactctatcgctccggttaactct
FAST14	agcgttctgagacatagagtcgtagatcggcagagcagaagagttaaccggagcgatag
FAST15	gactctatgtctcagaacgctaaacagttcctggaaatcaaaggtggttctcactcttg
FAST16	taccgatcagagcctggttagagttaccagagttagcgcaagagtgagaaccacctttg
FAST17	ccaggctctgatcggtaaaaaaggtgttgcttggatgaaacgtttcatggacaacgaca
FAST18	agttcgggttttcgcaagcgaaggtagagtaacgggtgtcgttgtccatgaaacgtttc

Primer	Sequence (5′→3′)
FAST19	gcttgcgaaaacccgaactctaccgctgtttctgacttccgtaccgctaactgctctct
FAST20	ttccagagagcagttagcggtac
TPA1	atgcaggaatctatcatccagtggcacggtgctaccaacacccgtgttccgttcggtat
TPA2	tacgctgctgttcctggtcagcgttagcggtgtcggtgtagataccgaacggaacacgg
TPA3	ccaggaacagcagcgtatctaccgtggtgaagtttggaactacctgtgcctggaatctg
TPA4	caccagcgaaggtggtacggaagtcaccagcacccgggatttcagattccaggcacagg
TPA5	taccaccttcgctggtgaaaccccgatagttgttgttcgtgacgctgaccaggaaatct
TPA6	atcagagcaccacggtgagcgcaacggttttcgaaagcgtagatttcctggtcagcgtc
TPA7	caccgtggtgctctgatagctctggaaaaatctggtcgtaccgactctttccagtgcgt
TPA8	cggtcaggtcaccctgacggttgtaagaccaagcgtggtaaacgcactggaaagagtcg
TPA9	cagggtgacctgaccggtgttgctttcgaaaaaggtgttaaaggtcagggtggtatgcc
TPA10	cgcagtttacgcggaccgtgttcttctttgcagaaagaagccggcataccaccctgacc
TPA11	ggtccgcgtaaactgcgtgttgctgttttctgcggtctggttttcggttctttctctga
TPA12	tccggacccaggtagtcttcgatagacggaacgtcttcagagaaagaaccgaaaaccag
TPA13	gactacctgggtccggaaatctgcgaacgtatcgaacgtgttctgcacaaaccggttga
TPA14	cagttgttcggcagtttctgggtgaaacgaccgataacttcaaccggtttgtgcagaac
TPA15	cagaaactgccgaacaactggaaactgtacttcgaaaacgttaaagactcttaccacgc
TPA16	tcagttcgaaggtggtgaagaacatgtgcagcagagaagcgtggtaagagtctttaacg
TPA17	cttcaccaccttcgaactgaaccgtctgtctcagaaaggtggtgttatcgttgacgaat
TPA18	cggtctatcatagagtaagaaacgtggtgaccaccagattcgtcaacgataacaccacc
TPA19	gtttcttactctatgatagaccgtggtgctaaagacgactcttacaaagaccaggctat
TPA20	gggtctttcagacggtaacgttcgttgtcagaacggatagcctggtctttgtaagagtc
TPA21	gttaccgtctgaaagacccgtctctgctggaaggtttcgaagaattcgaagacggtgtt
TPA22	gaacgaaacccgggaaaacagacagtatctgcagggtaacaccgtcttcgaattcttcg
TPA23	gttttcccgggtttcgttctgcagcagatacagaactctatcgctgttcgtcagctgct
TPA24	aggtaggtccagttcagttcagaagaagagatagatttcggcagcagctgacgaacagc
TPA25	gaactgaactggacctacctgggttacgctgacgactctgctgaacagcgtaaagttcg
TPA26	atgaaaccagccggacctatcaggttagcctgtttcagacgaactttacgctgttcagc
TPA27	ggtccggctggtttcatctctatggaagacggtgctgttggtggtttcgttcagcgtgg
TPA28	cccatttcgataacagcgtccaggttagcagcaccagcgataccacgctgaacgaaacc
TPA29	gacgctgttatcgaaatgggtggtgaccacgaaggttcttctgaaggtcgtgctaccga
TPA30	atgtgtttacggtaagctttccagaaaccacgaacagaggtttcggtagcacgaccttc
TPA31	tggaaagcttaccgtaaacacatgggtcaggaaatgcaggcttaaccaggagatatacc

Primer	Sequence (5′→3′)
TPA32	atcatggtatatctcctggttaagcc
TPA33	gcttaaccaggagatataccatgataaacgaaatccagatagctgctttcaacgctgct
TPA34	ggccactgttccatagcgtcagagtcgatggttttagcgtaagcagcgttgaaagcagc
TPA35	gctatggaacagtggccgaccttcttcaccaaagactgccactactgcgttaccaacgt
TPA36	caaacgataccagcagccagaccttcgtcgtggttgtcaacgttggtaacgcagtagtg
TPA37	ggctgctggtatcgtttgggctgactctcaggacatgctgaccgaccgtatctctgctc
TPA38	tgtgacggtaacggtgacgttcgtagatgttagcttcacgcagagcagagatacggtcg
TPA39	gtcaccgttaccgtcacatcctgggtctgccgtctatccagtctggtgacgctacccag
TPA40	gtgcatgatacgcagaaccatgaacggggtagaagcagaagcctgggtagcgtcaccag
TPA41	ggttctgcgtatcatgcacaccggtgaaaccgaagttttcgcttctggtgaatacctgg
TPA42	tcctgcagacgcagtttaccgtcgatggtggtgaatttgtccaggtattcaccagaagc
TPA43	gtaaactgcgtctgcaggaacgtatcgctgtttgcgactctaccgttaccgacaccctg
TPA44	catggtatatctcctgtcgacttacagcggcagagccatcagggtgtcggtaacgg
TPA45	aagtcgacaggagatataccatgaaccaccagatacacatccacgactctgacatcgc
TPA46	agcagcgtccagaacagactgacccggagcgcacgggaaagcgatgtcagagtcgtgg
TPA47	gtctgttctggacgctgctctgcaggctggtatcgaactgccgtactcttgccgtaaa
TPA48	cgtccagcagggtagaagcgcagttaccgcaagaacctttacggcaagagtacggcag
TPA49	cttctaccctgctggacggtaacatcgcttctttcaacggtatggctgttcgtaacga
TPA50	gcaaccgcacagcagaacctgttcagaagcgcacagttcgttacgaacagccataccg
TPA51	gttctgctgtgcggttgcaccgctgcttctgacatccgtatccacccgtcttctttcc
TPA52	gcggtgaaacgtttacgagcttccgggtccagacgacggaaagaagacgggtggatac
TPA53	gctcgtaaacgtttcaccgctaaagtttactctaacaccctggctgctccggacgttt
TPA54	tttagcacgtttaccaaccggcagacgcagacgcagcagagaaacgtccggagcagcc
TPA55	gccggttggtaaacgtgctaaattcgaagctggtcagtacctgctgatacacctggac
TPA56	cggcgggttagccatagagtaagaacgagattcaccgtcgtccaggtgtatcagcagg
TPA57	ctatggctaacccgccgcacgaatctgacggtatcaccctgcacgttcgtcacgttcc
TPA58	agatttcagctgctgaacgatggtagagaaacgaccacccggaacgtgacgaacgtgc
TPA59	ccatcgttcagcagctgaaatctggtgacaccctggacatcgaactgccgttcggttc
TPA60	gcatatcagcggacgagcgtcgtccggtttcagagcgatagaaccgaacggcagttcg
TPA61	gctcgtccgctgatatgcgttgctggtggtaccggtttcgctccgataaaatctgttc
TPA62	tcacgctgaactttacgtttagccaggtcgtccagaacagattttatcggagcgaaac
TPA63	ggctaaacgtaaagttcagcgtgacatcaccctgatatggggtgctcgtaacccgtct
TPA64	ctttacgccatttgtcgatagcagacggcaggtacagaccagacgggttacgagcacc

Primer	Sequence (5′→3′)
TPA65	ctgctatcgacaaatggcgtaaagtttggccgcagttccgttacatcgctgctatcac
TPA66	accagcgtgagcgtcagccggcatgtcacccaggtcggtgatagcagcgatgtaacgg
TPA67	ctgacgctcacgctggtcgtgttgacgacgctctgcgtacccacttcggtaacctgca
TPA68	accagagccggagaaccgcagcagtgaacaacgtggtcgtgcaggttaccgaagtggg
TPA69	ggttctccggctctggttcagtctgttcgtaccgctgcttctgacatgggtctgctgg
TPA70	gtcggaccggtagcgaaaacgtcagcgtggaagtcctgagccagcagacccatgtcag
TPA71	tcgctaccggtccgaccggtcaccactaagcatgcaggagatataccatg
TPA72	catggtatatctcctgcatgcttagtggtgaccg
TPA73	taagcatgcaggagatataccatgaccatcgttcaccgtcgtctggctctggctatcg
TPA74	gctttcagagcgatttccggaccgataccgtgcgggtcaccgatagccagagccagac
TPA75	ccggaaatcgctctgaaagctctgcagcagctgtctgttaccgaacgttctctgataa
TPA76	gcctgttccagagcagaccacggaccgtaaacttttatcagagaacgttcggtaacag
TPA77	gtctgctctggaacaggctgctcgtgtttgcgaaatggaaccgctgctgcaggacatc
TPA78	cactgaaccggctgggtcagggtaccagcttcttcgtgaacgatgtcctgcagcagcg
TPA79	cccagccggttcagtggggtgaaatcaccccgcaggctggtctgtctaccgttcagtc
TPA80	cttcaccgttttcgcaagcacggatagcagcggtagcagactgaacggtagacagacc
TPA81	gtgcttgcgaaaacggtgaagttgacgctgttatcgcttgcccgcaccacgaaaccgc
TPA82	agagacgggtaaccagagaaagcgataccagcacggtggatagcggtttcgtggtgcg
TPA83	ctttctctggttacccgtctctgctggctaacgttctgggtatgaacgaagaccaggt
TPA84	aacgatacgcagaccagcaccaaccagcatcaggaaaacctggtcttcgttcataccc
TPA85	gtgctggtctgcgtatcgttcacgttaccctgcacgaatctgttcgttctgctctgga
TPA86	gagcagcgttaacaaccagctgcggagacagacgttccagagcagaacgaacagattc
TPA87	gctggttgttaacgctgctcaggctgctgttcagacctgcaccctgctgggtgttccg
TPA88	agaagcgtgcgggttgataccgaaaacagcaactttcggtttcggaacacccagcagg
TPA89	gtatcaacccgcacgcttctgaaggtcagctgttcggtctggaagactctcagataac
TPA90	gaccacgtttacgcagggtttcaacagccggaacggttatctgagagtcttccagacc
TPA91	ccctgcgtaaacgtggtctggctgttgacggtccgatgggtgctgacatggttctggc
TPA92	tcgtgcagcatagcaacgtacaggtcgtgtttacgctgagccagaaccatgtcagcac
TPA93	gtacgttgctatgctgcacgaccagggtcacatcccgataaaactgctggctccgaac
TPA94	aacaacacgaccaccgatagacagagcagaagcaccgttcggagccagcagttttatc
TPA95	gtctatcggtggtcgtgttgttctgtcttctgttggtcacggttctgctatggacatc
TPA96	gcagcagagcggtagcgtcagcaacaccacgaccagcgatgtccatagcagaaccgtg
TPA97	gctaccgctctgctgcgtaccatcgctctgctgggtgctcagccggtttaa
TPA98	ttaaaccggctgagcacccagcagagcgatggtac

Table S5. List of protein coding sequences used in this study.

Proteins	Coding sequences
MHETase	MCAGGGSTPLPLPQQQPPQQEPPPPPVPLASRAACEALKDGNGDMVWP<br>NAATVVEVAAWRDAAPATASAAALPEHCEVSGAIAKRTGIDGYPYEIKF<br>RLRMPAEWNGRFFMEGGSGTNGSLSAATGSIGGGQIASALSRNFATIATD<br>GGHDNAVNDNPDALGTVAFGLDPQARLDMGYNSYDQVTQAGKAAVA<br>RFYGRAADKSYFIGCSEGGREGMMLSQRFPSHYDGIVAGAPGYQLPKA<br>GISGAWTTQSLAPAAVGLDAQGVPLINKSFSDADLHLLSQAILGTCDALD<br>GLADGIVDNYRACQAAFDPATAANPANGQALQCVGAKTADCLSPVQVT<br>AIKRAMAGPVNSAGTPLYNRWAWDAGMSGLSGTTYNQGWRSWWLGS<br>FNSSANNAQRVSGFSARSWLVDFATPPEPMPMTQVAARMMKFDFDIDPL<br>KIWATSGQFTQSSMDWHGATSTDLAAFRDRGGKMILYHGMSDAAFSAL<br>DTADYYERLGAAMPGAAGFARLFLVPGMNHCSGGPGTDRFDMLTPLVA<br>WVERGEAPDQISAWSGTPGYFGVAARTRPLCPYPQIARYKGSGDINTEA<br>NFACAAPP
MHETase^{M1}	MCAGGGSTPLPLPQQQPPQQEPPPPPVPLASRAACEALKAGNGDMVWP<br>NAATVVEVAAWRDAAPATASAAALPEHCEVSGAIAKRTGIDGYPYEIKF<br>RLRMPAEWNGRFFMEGGSGTNGSLTAATGSIGGGQIASALSRNFATIATD<br>GGHDNAVNDNPDALGTVAFGLDPQARLDMGYNSYDQVTQASKAAVAR<br>FYGRAADKSYFIGCSEGGREGMMLSQRFPSHYDGIVAGAPGYQLPKAGI<br>SGVWTTQSLAPAAVGLDAQGVPLINKSFSDADLHLLSQAILGTCDALDG<br>LADGIVDNYRACQAAFDPATAANPANGQALQCVGAKTADCLSPVQVTA<br>IQRAMAGPVNSAGTPLYNRWAWDAGMSGLTGTTYNQGWRSWWLGSF<br>NSSANNAQRVSGFSARSWLVDFATPPEPMPMTQVAARMMKFDFDIDPL<br>KIWATSGQFTQSSMDWLGATSTDLAAFRDRGGKMILYHGMSDAAFSAL<br>DTADYYERLGAAMPGAAGFARLYLVPGMNHCSGGPGTDRFDMLTPLVA<br>WVERGEAPDLISAWSGTPGYFGVAARTRPLCPYPQIARYKGSGDINTEAT<br>FACAAPP
MHETase^{M2}	MCAGGGSTPLPLPQQQPPQQEPPPPPVPLASRAACEALKDGNGDMVWP<br>NAATVVEVAAWRDAAPATASAAALPEHCEVSGAIARRTGIDGYPYEIKF<br>RLRMPAEWNGRFFMEGGSGTNGSLSAATGSIGGGQIASALSRNFATIATD<br>GGHDNAVNDNPDALGTVAFGLDPQARLDMGYNSYDQVTQASKAAVAR<br>FYGRAADKSYFIGSSEGGREGMMLSQRFPSHYDGIVAGAPGYQLPKAGI<br>SGAWTTQSLAPAAVGLDAQGVPLINKSFSDADLHLLSQAILGTCDALDG

Proteins	Coding sequences
	LADGIVDNYRACQAAFDPATAANPANGQALQCVGAKTADCLSPVQVTA<br>IKRAMAGPVNSAGTPLYNRWAWDAGMSGLSSTTYNQGWRSWWLGSF<br>NSSANNAQRVSGFSARSWLVDFATPPEPMPMTQVAARMMKFDFDIDPL<br>KIWATSGQFTQSSMDWHGATSTDLAAFRDRGGKMILYHGMSDAAFSAL<br>DTADYYERLGAAMPGAAGFARLFLVPGMDHCSGGPGTDRFDMLTPRVA<br>WVERGEAPDQISAWSGTPGYFGVAARTRPLCPYPQIARYKGSGDINTEA<br>NFACAAPP
MHETase^{M3}	MCAGGGSTPLPLPQQQPPQQEPPPPPVPLASRAACEALKDGNGDMVWP<br>NAATVVEVAAWRDAAPATASAAALPEHCEVSGAIAKRTGIDGYPYEIKF<br>RLRMPAVWNGRFFMEGGSGTNGSLSAATGSIGGGQIASALSRNFATIATD<br>GGHDNAVNDNPDALGTVAFGLGPQARLDMGYNSYDQVTQAGKAAVA<br>RFYGRAADKSYFIGCSEGGREGMMLSQRFPSHYDGIVAGAPGYQLPKA<br>GISGAWTTQSLAPAAVGLDAQGVPLINKSFSDADLHLLSQAILGTCDALD<br>GLAVGIVDNYRACQAAFDPATAANPANGQALQCVGAITADCLSPVQVTA<br>IKRAMAGPVNSAGTPLYNRWAWDAGMSGLSGSTYNQGWRSWWLGSF<br>DSSANNAQRVSGFSARSWLVDFATPPEPMPMTQVAARMMKFDFDIDPL<br>KIWATSGQFTQSSMDWHGASSTDLAAFRDRGGKMILYHGMSDAAFSAL<br>DTADYYERLGAAMPGAAGFARLFLVPGMNHCSGGPGTDRFDMLTPLVA<br>WVERGEAPDQISAWPGTPGYFGVAARTRPLCPYPQIARYKGSGDINTEA<br>NFACAAPP
FAST-PETase<br>(FastPETase)	MQTNPYARGPNPTAASLEASAGPFTVRSFTVSRPSGYGAGTVYYPTNAG<br>GTVGAIAIVPGYTARQSSIKWWGPRLASHGFVVITIDTNSTLDQPESRSS<br>QQMAALRQVASLNGTSSSPIYGKVDTARMGVMGWSMGGGGSLISAAN<br>NPSLKAAAPQAPWHSSTNFSSVTVPTLIFACENDSIAPVNSSALPIYDSMS<br>QNAKQFLEIKGGSHSCANSGNSNQALIGKKGVAWMKRFMDNDTRYSTF<br>ACENPNSTAVSDFRTANCSLE
BmoR	MSKMQEFARLETVASMRRAVWDGNECQPGKVADVVLRSWTRCRAEG<br>VVPNARQEFDPIPRTALDETVEAKRALILAAEPVVDALMEQMNDAPRMI<br>ILNDERGVVLLNQGNDTLLEDARRRAVRVGVCWDEHARGTNAMGTAL<br>AERRPVAIHGAEHYLESNTIFTCTAAPIYDPFGEFTGILDISGYAGDMGPV<br>PIPFVQMAVQFIENQLFRQTFADCILLHFHVRPDFVGTMREGIAVLSREGT<br>IVSMNRAGLKIAGLNLEAVADHRFDSVFDLNFGAFLDHVRQSAFGLVRV<br>SLYGGVQVYARVEPGLRVPPRPAAHARPPRPAPRPLDSLDTGDAAVRLAI<br>DRARRAIGRNLSILIQGETGAGKEVFAKHLHAESPRSKGPFVAVNCAAIP

Proteins	Coding sequences
	EGLIESELFGYEEGAFTGGRRKGNIGKVAQAHGGTLFLDEIGDMAPGLQ<br>TRLLRVLQDRAVMPLGGREPMPVDIALVCATHRNLRSLIAQGQFREDLY<br>YRLNGLAISLPPLRQRSDLAALVNHILFQCCGGEPHYSVSPEVMTLFKRH<br>AWPGNLRQLHNVLDAALAMLDDGHVIEPHHLPEDFVMEVDSGLRPIEE<br>DGSTAAHRARQPASGSGPAKKLQDLALDAIEQAIEQNEGNISVAARQLG<br>VSRTTIYRKLRQLSPTGCHRPAHWSQSRIGT
BmoR^{N207S}	MSKMQEFARLETVASMRRAVWDGNECQPGKVADVVLRSWTRCRAEG<br>VVPNARQEFDPIPRTALDETVEAKRALILAAEPVVDALMEQMNDAPRMI<br>ILNDERGVVLLNQGNDTLLEDARRRAVRVGVCWDEHARGTNAMGTAL<br>AERRPVAIHGAEHYLESNTIFTCTAAPIYDPFGEFTGILDISGYAGDMGPV<br>PIPFVQMAVQFIESQLFRQTFADCILLHFHVRPDFVGTMREGIAVLSREGT<br>IVSMNRAGLKIAGLNLEAVADHRFDSVFDLNFGAFLDHVRQSAFGLVRV<br>SLYGGVQVYARVEPGLRVPPRPAAHARPPRPAPRPLDSLDTGDAAVRLAI<br>DRARRAIGRNLSILIQGETGAGKEVFAKHLHAESPRSKGPFVAVNCAAIP<br>EGLIESELFGYEEGAFTGGRRKGNIGKVAQAHGGTLFLDEIGDMAPGLQ<br>TRLLRVLQDRAVMPLGGREPMPVDIALVCATHRNLRSLIAQGQFREDLY<br>YRLNGLAISLPPLRQRSDLAALVNHILFQCCGGEPHYSVSPEVMTLFKRH<br>AWPGNLRQLHNVLDAALAMLDDGHVIEPHHLPEDFVMEVDSGLRPIEE<br>DGSTAAHRARQPASGSGPAKKLQDLALDAIEQAIEQNEGNISVAARQLG<br>VSRTTIYRKLRQLSPTGCHRPAHWSQSRIGT
BmoR$^{F177L/Q285L}$	MSKMQEFARLETVASMRRAVWDGNECQPGKVADVVLRSWTRCRAEG<br>VVPNARQEFDPIPRTALDETVEAKRALILAAEPVVDALMEQMNDAPRMI<br>ILNDERGVVLLNQGNDTLLEDARRRAVRVGVCWDEHARGTNAMGTAL<br>AERRPVAIHGAEHYLESNTIFTCTAAPIYDPFGELTGILDISGYAGDMGPV<br>PIPFVQMAVQFIENQLFRQTFADCILLHFHVRPDFVGTMREGIAVLSREGT<br>IVSMNRAGLKIAGLNLEAVADHRFDSVFDLNFGAFLDHVRLSAFGLVRV<br>SLYGGVQVYARVEPGLRVPPRPAAHARPPRPAPRPLDSLDTGDAAVRLAI<br>DRARRAIGRNLSILIQGETGAGKEVFAKHLHAESPRSKGPFVAVNCAAIP<br>EGLIESELFGYEEGAFTGGRRKGNIGKVAQAHGGTLFLDEIGDMAPGLQ<br>TRLLRVLQDRAVMPLGGREPMPVDIALVCATHRNLRSLIAQGQFREDLY<br>YRLNGLAISLPPLRQRSDLAALVNHILFQCCGGEPHYSVSPEVMTLFKRH<br>AWPGNLRQLHNVLDAALAMLDDGHVIEPHHLPEDFVMEVDSGLRPIEE<br>DGSTAAHRARQPASGSGPAKKLQDLALDAIEQAIEQNEGNISVAARQLG<br>VSRTTIYRKLRQLSPTGCHRPAHWSQSRIGT

Proteins	Coding sequences
$TphA1_{II}$ UniProt ID: Q3C1D2	MNHQIHIHDSDIAFPCAPGQSVLDAALQAGIELPYSCRKGSCGNCASTLLD GNIASFNGMAVRNELCASEQVLLCGCTAASDIRIHPSSFRRLDPEARKRFT AKVYSNTLAAPDVSLLRLRLPVGKRAKFEAGQYLLIHLDDGESRSYSMA NPPHESDGITLHVRHVPGGRFSTIVQQLKSGDTLDIELPFGSIALKPDDARP LICVAGGTGFAPIKSVLDDLAKRKVQRDITLIWGARNPSGLYLPSAIDKWR KVWPQFRYIAAITDLGDMPADAHAGRVDDALRTHFGNLHDHVVHCCGSP ALVQSVRTAASDMGLLAQDFHADVFATGPTGHH
$TphA2_{II}$ UniProt ID: Q3C1D5	MQESIIQWHGATNTRVPFGIYTDTANADQEQQRIYRGEVWNYLCLESEIPG AGDFRTTFAGETPIVVVRDADQEIYAFENRCAHRGALIALEKSGRTDSFQC VYHAWSYNRQGDLTGVAFEKGVKGQGGMPASFCKEEHGPRKLRVAVFCG LVFGSFSEDVPSIEDYLGPEICERIERVLHKPVEVIGRFTQKLPNNWKLYFE NVKDSYHASLLHMFFTTFELNRLSQKGGVIVDESGGHHVSYSMIDRGAK DDSYKDQAIRSDNERYRLKDPSLLEGFEEFEDGVTLQILSVFPGFVLQQIQ NSIAVRQLLPKSISSSELNWTYLGYADDSAEQRKVRLKQANLIGPAGFISM EDGAVGGFVQRGIAGAANLDAVIEMGGDHEGSSEGRATETSVRGFWKAY RKHMGQEMQA
$TphA3_{II}$ UniProt ID: Q3C1D4	MINEIQIAAFNAAYAKTIDSDAMEQWPTFFTKDCHYCVTNVDNHDEGLA AGIVWADSQDMLTDRISALREANIYERHRYRHILGLPSIQSGDATQASASTP FMVLRIMHTGETEVFASGEYLDKFTTIDGKLRLQERIAVCDSTVTDTLMAL PL
$TphB_{II}$ UniProt ID: Q3C1D3	MTIVHRRLALAIGDPHGIGPEIALKALQQLSVTERSLIKVYGPWSALEQAA RVCEMEPLLQDIVHEEAGTLTQPVQWGEITPQAGLSTVQSATAAIRACENG EVDAVIACPHHETAIHRAGIAFSGYPSLLANVLGMNEDQVFLMLVGAGLRI VHVTLHESVRSALERLSPQLVVNAAQAAVQTCTLLGVPKPKVAVFGINPH ASEGQLFGLEDSQITVPAVETLRKRGLAVDGPMGADMVLAQRKHDLYVA MLHDQGHIPIKLLAPNGASALSIGGRVVLSSVGHGSAMDIAGRGVADATA LLRTIALLGAQPV
PobA***	MKTQVAIIGAGPSGLLLGQLLHKAGIDNVILERQTPDYVLGRIRAGVLEQG MVDLLREAGVDRRMARDGLVHEGVEIAFAGQRRRIDLKRLSGGKTVTVY GQTEVTRDLMEAREACGATTVYQAAEVRLHDLQGERPYVTFERDGERLR LDCDYIAGCDGFHGISRQSIPAERLKVFERVYPFGWLGLLADTPPVSHELIY ANHPRGFALCSQRSATRSRYYVQVPLSEKVEDWSDERFWTELKARLPSEV AEKLVTGPSLEKSIAPLRSFVVEPMQHGRLFLAGDAAHIVPPTGAKGLNLA ASDVSTLYRLLLKAYREGRGELLERYSAICLRRIWKAERFSWWMTSVLHR FPDTDAFSQRIQQTELEYYLGSEAGLATIAENFVGLPYEEIE

Table S6. PCR and plasmids construction details.

Plasmids	Templates	Fragments	Primers	Length/bp
pEG1	—	sfGFP	lm1 + lm2	763
	pYH1[5]	Backbone1	lm3 + lm4	5110
pEG2	Plasmid#1[6]	SacB	lm7 + lm8	1463
	pEG1	Backbone2	lm9 + lm10	5855
—	pEG2	ep-PCR1	BmoR1 + BmoR4	1157
		ep-PCR2	BmoR1 + BmoR6	2040
		Backbone3	BmoR2 + BmoR3	6163
		Backbone4	BmoR2 + BmoR5	5279
pEG5	—	p15A	lm39 + lm40	557
	pEG3	Fragment1	lm41 + lm42	1422
	p15A, Fragment1	Fragment2	lm39 + lm42	1962
	pEG3	Backbone5	lm43 + lm44	5302
pEG6	—	p15A	lm39 + lm40	557
	pEG4	Fragment3	lm41 + lm42	1422
	p15A, Fragment3	Fragment4	lm39 + lm42	1962
	pEG4	Backbone6	lm43 + lm44	5302
pLM1	—	MHETase	MHET1−MHET44	1761
	pET-28a(+)	Backbone7	lm15 + lm16	5241
pLM2	pLM1	Fragment5	lm19 + lm20	7011
pLM3	—	FastPETase	FAST1−FAST20	795
	pET-28a(+)	Backbone8	lm23 + lm24	5262
pLM4	pLM1	M1	lm26 + lm27	1791
	pLM3	F1	FAST20 + lm28	829
	M1, F1	M1-F1	lm26 + FAST20	2601
	pLM1	Backbone9	lm15 + lm23	5261
pLM5	pLM4	Fragment6	lm19 + lm20	7811

Plasmids	Templates	Fragments	Primers	Length/bp
pLM6	pLM5	M2	lm30 + lm32	1789
	pLM5	F2	lm31 + lm34	828
	M2, F2	F2-M2	lm32 + lm34	2598
	pLM4	Backbone10	lm24 + lm33	5261
pLM7	pLM6	Fragment7	lm19 + lm20	7811
pLM8	—	SUMO	lm35 + lm36	313
	pLM4	M3	MHET1 + MHET44	1761
	SUMO, M3	S-M3	lm35 + MHET44	2059
	pLM4	Backbone11	lm16 + lm37	5242
pLM9	pLM8	Fragment8	lm19 + lm20	7251
pLM10	M1, F1	M1-F1	lm26 + FAST20	2601
	pLM8	Backbone12	lm23 + lm 36	5555
pLM11	pLM10	Fragment9	lm19 + lm20	8105
pLM12	M2, F2	F2-M2	lm32 + lm34	2598
	pLM8	Backbone13	lm33 + lm38	5564
pLM13	pLM12	Fragment10	lm19 + lm20	8105
—	pLM11	ep-PCR3	SMF1 + SMF4	1852
		Backbone14	SMF2 + SMF3	6322
pEG7	pLM11	Kan	lm56 + lm57	816
	pEG3	Backbone15	lm58 + lm59	6467
pLM16	pLM11	Fragment11	D40A-F + D40A-R	8152
pLM17		Fragment12	K84R-F + K84R-R	
pLM18		Fragment13	E104V-F + E104V-R	
pLM19		Fragment14	S122T-F + S122T-R	
pLM20		Fragment15	D169G-F + D169G-R	
pLM21		Fragment16	G188S-F + G188S-R	
pLM22		Fragment17	C208S-F + C208S-R	
pLM23		Fragment18	A246V-F + A246V-R	

Plasmids	Templates	Fragments	Primers	Length/bp
pLM24		Fragment19	D295V-F + D295V-R	
pLM25		Fragment20	K328I-F + K328I-R	
pLM26		Fragment21	K342Q-F + K342Q-R	
pLM27		Fragment22	S370T-F + S370T-R	
pLM28		Fragment23	G371S-F + G371S-R	
pLM29		Fragment24	T372S-F + T372S-R	
pLM30		Fragment25	N387D-F + N387D-R	
pLM31	pLM11	Fragment26	H451L-F + H451L-R	8152
pLM32		Fragment27	T454S-F + T454S-R	
pLM33		Fragment28	F505Y-F + F505Y-R	
pLM34		Fragment29	N511D-F + N511D-R	
pLM35		Fragment30	L528R-F + L528R-R	
pLM36		Fragment31	Q540L-F + Q540L-R	
pLM37		Fragment32	S545P-F + S545P-R	
pLM38		Fragment33	N580T-F + N580T-R	
pLM39	—	$TphA2_{II}$	TPA1 − TPA32	1761
	—	$TphA3_{II}$	TPA33 − TPA44	506
	—	$TphA1_{II}$	TPA45 − TPA70	1053
	—	$TphB_{II}$	TPA71 − TPA98	969
	$TphA2_{II}$, $TphA3_{II}$, $TphA1_{II}$, $TphB_{II}$	$TphAB_{II}$	TPA1 − TPA98	3716
	—	Backbone16	lm60 + lm61	3196
pLM40	pLM39	$TphAB_{II}$	TPA1−TPA98	3716
	—	Backbone17	lm60 + lm61	3176
pLM41	pLM39	$TphAB_{II}$	TPA1−TPA98	3716
	—	Backbone18	lm60 + lm61	3166
pLM42	pZE-PobA(Y385F/T294A/V349A)[7]	PobA***	lm64 + lm65	1185
	pLM39	Backbone19	lm66 + lm67	3198

Plasmids	Templates	Fragments	Primers	Length/bp
pLM43	pZE-PobA(Y385F/T294A/V349A)[7]	PobA***	lm64 + lm65	1185
	pLM40	Backbone20	lm66 + lm67	3178
pLM44	pZE-PobA(Y385F/T294A/V349A)[7]	PobA***	lm64 + lm65	1185
	pLM41	Backbone21	lm66 + lm67	3168
pLM45	pLM43	Fragment34	lm65 + lm68	1212
	pLM40	Backbone22	lm69 + lm70	6899

References

[1] J. Zhang, H. Wang, Z. Luo, Z. Yang, Z. Zhang, P. Wang, M. Li, Y. Zhang, Y. Feng, D. Lu, Y. Zhu, *Commun. Biol*. **2023**, 6, 1135.

[2] B. C. Knott, E. Erickson, M. D. Allen, J. E. Gado, R. Graham, F. L. Kearns, I. Pardo, E. Topuzlu, J. J. Anderson, H. P. Austin, G. Dominick, C. W. Johnson, N. A. Rorrer, C. J. Szostkiewicz, V. Copié, C. M. Payne, H. L. Woodcock, B. S. Donohoe, G. T. Beckham, J. E. McGeehan, *Proc. Natl. Acad. Sci.* **2020**, 117, 25476.

[3] A. V. Pinto, P. Ferreira, R. P. P. Neves, P. A. Fernandes, M. J. Ramos, A. L. Magalhães, *ACS Catal.* **2021**, 11, 10416.

[4] A. Rauwerdink, R. J. Kazlauskas, *ACS Catal.* **2015**, 5, 6153.

[5] H. Yu, Z. Chen, N. Wang, S. Yu, Y. Yan, Y.-X. Huo, *Metab. Eng.* **2019**, 56, 28.

[6] C. Huang, L. Guo, J. Wang, N. Wang, Y.-X. Huo, Appl. *Microbiol. Biotechnol.* **2020**, 104, 7943.

[7] Z. Chen, T. Chen, S. Yu, Y.-X. Huo, Biotechnol. *Biofuels Bioprod.* **2022**, 15, 43.
